# Alaskan carbon-climate feedbacks will be weaker than inferred from short-term experiments

**DOI:** 10.1038/s41467-020-19574-3

**Published:** 2020-11-16

**Authors:** Nicholas J. Bouskill, William J. Riley, Qing Zhu, Zelalem A. Mekonnen, Robert F. Grant

**Affiliations:** 1 Climate and Ecosystem Sciences Division, Lawrence Berkeley National Laboratory, Berkeley, CA 94720 USA; 2 Department of Renewable Resources, University of Alberta, Edmonton, Canada

**Keywords:** Carbon cycle, Cryospheric science, Ecological modelling

## Abstract

Climate warming is occurring fastest at high latitudes. Based on short-term field experiments, this warming is projected to stimulate soil organic matter decomposition, and promote a positive feedback to climate change. We show here that the tightly coupled, nonlinear nature of high-latitude ecosystems implies that short-term (<10 year) warming experiments produce emergent ecosystem carbon stock temperature sensitivities inconsistent with emergent multi-decadal responses. We first demonstrate that a well-tested mechanistic ecosystem model accurately represents observed carbon cycle and active layer depth responses to short-term summer warming in four diverse Alaskan sites. We then show that short-term warming manipulations do not capture the non-linear, long-term dynamics of vegetation, and thereby soil organic matter, that occur in response to thermal, hydrological, and nutrient transformations belowground. Our results demonstrate significant spatial heterogeneity in multi-decadal Arctic carbon cycle trajectories and argue for more mechanistic models to improve predictive capabilities.

## Abstract (web summary)

Warming in the high latitudes is expected to stimulate soil organic matter decomposition which leads to enhanced carbon emissions. Here, the authors show that short-term experiments do not capture the complexity of vegetation dynamics in the Arctic and might thus not provide a full picture of long term processes.

## Introduction

Land carbon-climate feedbacks represent significant uncertainty in predicting atmospheric carbon concentrations under a changing climate^1^. Permafrost soils contain more carbon between 0 and 3 m depth^2,3^ (1035 ± 150 PgC) than in the current atmosphere. These soils are warming at twice the global average (0.6 °C per decade)^4^, and empirical observations and model predictions indicate that this warming leads to a consistent release of greenhouse gases (i.e., CO_2_ and CH_4_) from soils, leading to a positive feedback to climate change^5^.

Current understanding of the land carbon-climate feedback over the 21st century is predominantly based upon laboratory incubations^6^ and short- (1–5 years) or medium-term (5–20 year) warming studies^7–10^. High-latitude field-based warming experiments have commonly used open top chambers (OTCs) to warm near-surface air temperatures, and have shown a general increase in chamber air temperatures (up to 2.1 °C)^11^, and shifts in aboveground biomass and community composition^12^. Changes in vegetation, broadly favoring vascular plants (shrubs in particular) over non-vascular plants (e.g., lichen and bryophytes^13,14^) and deciduous shrubs over evergreen shrubs, are generally attributable to shifting thermal niches^15^, altered plant phenology^12^, and elevated mineralization rates increasing plant nutrient availability^16^ in these nitrogen-limited habitats. The subsequent increase in productivity can result in increased plant carbon fixation, belowground allocation, and soil organic carbon (SOC) stocks^17^.

SOC gains through enhanced vegetation growth^17^ can be offset through elevated microbial decomposition under warming^6,18^. Rusted et al.^16^ performed a meta-analysis of the responses of tundra respiration to short-term warming, and reported a 20% increase in tundra respiration under short-term warming, while recent ecosystem manipulation experiments focusing on winter and summer warming have demonstrated carbon effluxes via heterotrophic respiration outweigh inputs via plant productivity as the active layer depth increases^18^. However, the microbial community response to warming is complex and depends on concurrent changes in soil hydrology. For example, while drying or anoxic microsite formation under saturating conditions can reduce microbial activity^6,9^, a slight decrease in soil moisture has also been shown to increase microbial activity^19^.

Multi-decadal predictions based on short-term experiments assume some degree of consistency in ecosystem response in order to extrapolate a potential trend across longer time scales. However, medium-term (i.e., 20 year) field experiments have demonstrated that short-term ecosystem responses to perturbation can be inconsistent with longer-term responses^20,21^. In one of the longest running tundra warming experiments, Sistla et al.^8^ reported no change in soil carbon stocks over 20 years of warming, despite increasing plant biomass and woody dominance, increasing wintertime temperatures, and suppressed surface decomposer activity. Interestingly, this study also showed that the most dynamic response to warming occurred deeper in the soil, with clear changes in microbial activity and mineral soil carbon stocks.

Several studies have highlighted a strong non-linearity of ecosystem responses to perturbation^22^. Mechanistic modeling approaches can be employed to examine the influence of ecosystem processes that play out on different time scales, but determine the long-term fate of soil carbon. For example, both observations and model predictions demonstrate the significance of a long-term deepening active layer^23^ and its effects on soil hydrology, soil thermal conductivity, and nutrient availability^24^. Furthermore, complex interactions between and among plant and microbial communities, including physiological adaptation to changing climate^25^, can lead to non-linear climate feedbacks. A hypothetical example of such non-linear feedbacks would be transitions of net CO_2_ fluxes between sinks and sources as different members of an ecosystem responded to perturbation. Initial warming can stimulate fast-growing microbial decomposers before other components of the ecosystem^6,18^. Their activity could tip an ecosystem towards becoming a short-term source of CO_2_ to the atmosphere. However, over time, a warmer environment with higher atmospheric CO_2_, increased precipitation, and sufficient nutrient availability can promote plant productivity^17^. Over a sufficiently long period of time, increased vegetation growth can transition the ecosystem towards becoming carbon neutral, or even a carbon sink.

In this work we apply a well-tested mechanistic land model, *ecosys* (see Methods section for details), to examine differences in ecosystem carbon cycle responses between observed and modeled short-term (<10 year) warming experiments and modeled long-term (100 year) changes under 21st century expected temperature, precipitation, and CO_2_ concentrations. Through this approach, we show that short-term warming resulted in a much higher rate of soil carbon loss relative to multi-decadal responses. This can partly be attributed to long-term perturbation occurring at a lower rate of change. However, the short-term warming experiments favor heterotrophic activity, and hence soil carbon loss, and generally are not designed to capture longer-term, non-linear dynamics of vegetation, that occur in response to thermal, hydrological, and nutrient transformations belowground. Herein, we discuss the specific mechanisms regulating these feedbacks.

## Results

Our modeling experiment focus on four sites representative of distinct ecosystems across Alaska in which experimental manipulations have been performed (Methods section). Utqiaġvik (formerly Barrow, 71°18′N, 156°40′W) lies 3 m above sea level (a.s.l) on the northern edge of the Alaskan Arctic Coastal Plain. It is underlain by continuous permafrost and characterized by short, cool growing seasons. Toolik (68°38′N, 149°34′W. 740 m a.s.l), in the foothills of the Brooks Range, is also underlain by continuous permafrost, but with comparatively warmer summers. Eight Mile Lake (63°52′N, 149°13′W. 670 m a.s.l) is a moist acidic tundra site with discontinuous permafrost located in the northern foothills of the Alaska Range. Finally, Delta Junction (63°55′N, 145°44′W. ~1100 a.s.l) is an upland boreal forest ecosystem with heterogeneous coverage of deep discontinuous permafrost. All four sites have hosted passive warming experiments^7,9,26^, which we leverage here to infer emergent temperature sensitivity of decomposition and to evaluate *ecosys*.

### Model testing against perturbation experiments

**Fig. 1:**
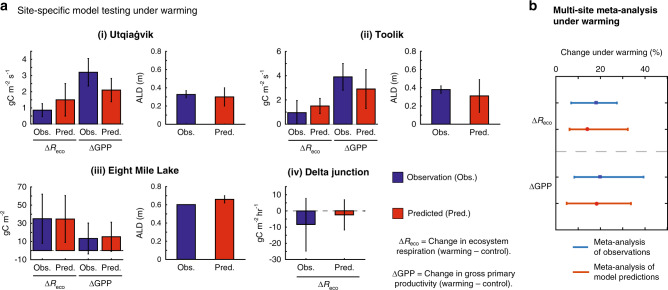
Model-data comparisons demonstrate *ecosys* accurately represents ecosystem responses to short-term warming. **a** Site-specific comparisons between observations and *ecosys* predictions for (i) Utqiaġvik, (ii) Toolik, (iii) Eight Mile Lake, and (iv) Delta Junction. The model output was collected over the same time period as that of the experimental perturbation and observations. Experimental data were taken from refs. ^7,9,26,60,75^ (see Table 1), while active layer depth was aggregated from the circumpolar active layer monitoring network (https://www2.gwu.edu/~calm/) for Utqiaġvik and Toolik, and compiled from measurements at Eight Mile Lake. The change in ecosystem respiration (ΔR_eco_) and gross primary productivity (ΔGPP) represent the difference between warming and the ambient control experiments. These data were extracted from the experimental publications. The active layer depth is a direct comparison between measurements and simulations. Each bar shows the interannual mean of each metric, and the error bars represent the standard deviation around the mean. Two-way analysis of variance tests indicated no significant differences between observed and modeled perturbation responses. **b** Meta-analysis of model output compared with a recent meta-analysis of observations^27^ of high-latitude response to warming at the four sites. This forest plot shows the % divergence from the control studies for both observations (in blue), and the modeled response (in red). Each bar depicts the mean (solid square), and the variance (standard deviation) around the mean. A one-way anova found no significant differences between the % divergence between the model output and the empirical data.

Across the four sites (Fig. 1a) and against a recently compiled meta-analysis including the four sites^27^ (Fig. 1b), *ecosys* accurately reproduced the observed active layer depth (ALD) and responses of gross primary productivity (GPP) and ecosystem respiration (i.e., heterotrophic + autotrophic respiration, R_eco_) to short-term warming (Fig. 1 and Supplementary Table 4). These results, and previously described evaluations against high-latitude diurnal, seasonal, and interannual variability of C and energy dynamics^14,24,28^, demonstrate that *ecosys* provides a reasonable representation of the response of high-latitude ecosystems to perturbation and can be extended to the model experiments described below.

### Modeled ecosystem responses to short-term warming

**Fig. 2:**
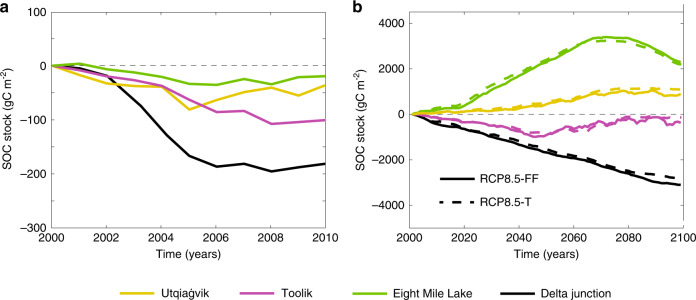
Soil organic carbon stock trajectories differ between short and long-term warming. Soil organic stock trajectories under **a** short-term warming and **b** long-term perturbation simulations (warming only (RCP8.5-T) and fully forced (RCP8.5-FF)) for the 0–3 m soil profile. Short- and long-term SOC stock trajectories have been normalized to unperturbed simulations.

Under short-term warming manipulations we found a clear increase in shallow (~0.1 m) soil temperature at each site (Average increase: Utqiaġvik +0.85 °C; Toolik +1.1 °C; Eight Mile Lake +0.3 °C; and Delta Junction +0.9 °C), and air temperature, and a slight decline in soil moisture (Supplementary Table 3). Soil nutrient concentrations were largely unaffected, likely due to microbial biomass nitrogen and phosphorus increasing at each site (Supplementary Table 3). Modeled whole soil profile SOC stocks declined at all four sites (Fig. 2a). The extent of this 10-year decline differed by site, with SOC loss being highest at Delta Junction (net carbon loss of 0.18 kg C m^−2^), followed by Toolik (0.09 kg C m^−2^), Utqiaġvik (0.05 kg C m^−2^), and Eight Mile Lake (0.02 kg C m^−2^).

### Modeled ecosystem responses to long-term warming

Under long-term perturbation, air temperature rose at all sites over time at the same rate under RCP8.5-T and RCP8.5-FF (Supplementary Table 4). Soil moisture also increased at all sites, although the increase was larger under RCP8.5-FF than RCP8.5-T due to a simulated rise in precipitation under RCP8.5-FF. Elevated soil water content increased soil thermal conductance, resulting in a deepening ALD modeled at all tundra sites. At Eight Mile Lake the ALD increased by over 1 m concurrent with a small increase in soil moisture across the soil profile (Supplementary Table 2). Delta Junction, which is not underlain by permafrost, also had a large increase in soil moisture under both perturbation scenarios (~28 and 70% increase within surface soils, and ~40% at 1 m under RCP8.5-T and RCP8.5-FF, respectively). The ALDs at Utqiaġvik and Toolik doubled between 2000 and 2100 to 0.52 (±0.8) and 0.60 m (±0.88, mean and standard deviation), respectively.

Long-term modeled SOC stock responses were more complex than in the short-term simulations (Fig. 2b). The trajectories of SOC stocks were most similar between the short- and long-term perturbations at Delta Junction and Toolik (both sites lost SOC by 2100; Fig. 2a, b). Toolik SOC stocks decreased from 2000 to 2050 before increasing from 2050 to 2100 under RCP8.5-FF (Fig. 2b). At Toolik, net SOC losses over 100 years were ~0.2 kg C m^−2^ (0.03% of baseline stocks) and ~0.4 kg C m^−2^ (0.06% of baseline stocks) for RCP8.5-T and RCP8.5-FF simulations, respectively. Delta Junction, however, showed a monotonic decrease in SOC between 2000 and 2100, resulting in an overall SOC loss of 3 and 3.4 kg C m^−2^ (0.48 and 0.42% of baseline stocks) for RCP8.5-T and RCP8.5-FF simulations, respectively.

At Eight Mile Lake and Utqiaġvik, the modeled SOC differed between short-term and long-term warming scenarios. Modeled SOC accumulation at Eight Mile Lake peaked around the year 2070 and declined thereafter. However, Eight Mile Lake remained a carbon sink by 2100, with net SOC stock increases of 2.1 and 2.15 kg C m^−2^ (0.38 and 0.39% of baseline stocks) under RCP8.5-FF and RCP8.5-T simulations, respectively. By comparison, the SOC accumulation rate at Utqiaġvik was lower and monotonic (Fig. 2b). Nevertheless, modeled Utqiaġvik SOC stocks increased by 0.7 kg C m^−2^ and 1 kg C m^−2^ (0.12 and 0.17% of baseline stocks) under RCP8.5-FF and RCP8.5-T simulations, respectively. Finally, over the 21st century, the contribution of wintertime respiration increased significantly, from a two-fold at Utqiaġvik, to a six-fold increase at Delta Junction (Supplementary Table 3).

### Emergent SOC temperature sensitivity under short- and long-term warming

We defined a simple depth-dependent emergent temperature sensitivity metric (*ν*) to compare SOC stock changes under different forcings. This metric (*ν*) represents changes in SOC stocks (g C m^−2^) normalized to changes in soil temperature (°C) at specific soil depths (0.1, 0.6, 1.0, and 1.7 m) over the experiment length (10 or 100 years):

1 $$\nu = \frac{{\left( {C_{\mathrm{p}} - C_{\mathrm{b}}} \right)/{\Delta}T}}{{{\Delta}t}}$$

where *C*_p_ and *C*_b_ represent SOC stocks under perturbed and baseline conditions, respectively, at specific depths. Δ*T* represents the change in temperature under short-term or long-term warming experiments at specific depths, and Δ*t* represents the simulation period.

**Fig. 3:**
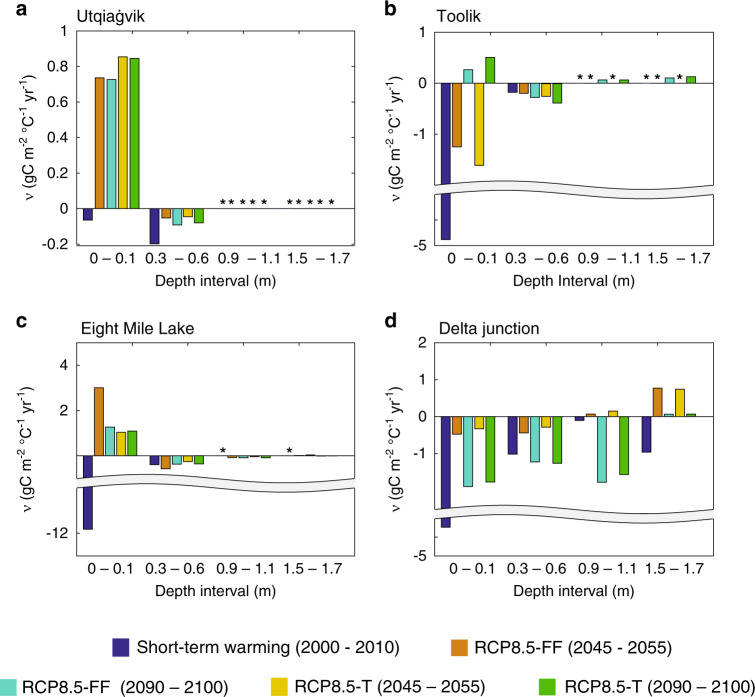
The magnitude and sign of the emergent temperature sensitivities varies between sites, short-term and long-term warming, and depth. The figure shows the modeled depth-resolved emergent temperature sensitivity (ν) of soil organic carbon at each **a** Utqiaġvik, **b** Toolik, **c** Eight Mile Lake, and **d** Delta Junction, under short-term and the two long-term (i.e., RCP8.5-T and RCP8.5-FF) scenarios across two time periods (2045–2055 and 2090–2100). A positive *ν* value denotes a period where the ecosystem is acting as a sink for carbon, while a negative value indicates the ecosystem is losing carbon. Asterisk denotes periods where permafrost was simulated to occur at that depth, and therefore no change in soil organic carbon stock occurred.

The emergent temperature sensitivity of SOC is clearly different between short- and long-term responses at each site (Fig. 3). In many cases, *ν* is much larger under short-term than long-term warming (note the broken *y*-axis range in Fig. 3a–c) within the most biologically active top 0.1 m of soil. At Utqiaġvik and Eight Mile Lake, the sign of *ν* reverses between the short- and long-term response. These sites are a source of carbon under short-term warming, and transition to carbon sinks over a 50–100 year time period (Fig. 2). Toolik *ν* values switch signs between the first (2000–2050) and last (2050–2100) 50-year periods of warming. Finally, there is a clear depth dependency to the calculated temperature sensitivity value under long-term (100 year) warming. For example, Utqiaġvik, Toolik, and Eight Mile Lake have opposite signs, and much smaller values, of *ν* in the 0.3−0.6 depth range compared to the top 0.1 m (Fig. 3a–c). Both Toolik and the boreal Delta Junction sites have a small accumulation of SOC at depths >1.5 m by 2100 (Fig. 3b, d).

## Discussion

### Short-term warming

Observational experiments are invaluable for improving conceptual understanding of the mechanisms underpinning ecosystem responses to climate change. In this study we have examined how these short-term responses might evolve when considered within the context of multi-decadal climate change forcing controlled by responses that emerge over longer time scales. Notwithstanding qualitative similarities between the trajectory of SOC stocks at Toolik and Delta Junction under short-term and long-term perturbation, we generally noted very different emergent SOC stock temperature sensitivities in four diverse Alaskan ecosystems. While the short-term SOC sensitivities were driven primarily by the rapid response of temperature-sensitive microorganisms, the long-term sensitivities were associated with changing vegetation dynamics and greater SOC turnover below surface soils (i.e., >0.1 m).

The short-term warming scenarios are akin to a stepwise experiment, imposing a higher rate of soil warming (average: ~0.7 °C, range: 0.3–1.3 °C) relative to the long-term perturbation experiments over the first 10 years (average: 0.2 °C, range: 0–0.5 °C). This aggressive short-term warming scenario stimulated heterotrophic microbial functional guilds (which have strong thermodynamic controls on their activity) more rapidly than vegetation, consistent with observations^6,18,29^ (although see Deane-Coe et al.^30^). Respiration rates can also increase due to a modeled decline in soil moisture under short-term warming. A small decline in soil moisture can increase oxygen diffusion into soils which can further stimulate heterotrophic and autotrophic respiration^19^. The rapidity with which belowground activity increases tends to exceed vegetation responses and leads to a net decline in SOC. This difference is best demonstrated by the emergent SOC temperature sensitivity of the top 0.1 m of soil, which is several times larger under short-term than long-term perturbation (Fig. 3).

Under short-term warming a small, but not statistically significant, increase in vegetation carbon of 5–24% was modeled after 10 years. This increase was primarily driven by increased shrub biomass, with little to no change in the biomass of other plant functional types at each site. Furthermore, despite an overall loss of soil carbon, litter carbon increased at all sites concurrently with increasing plant biomass, consistent with previous results^13^.

**Fig. 4:**
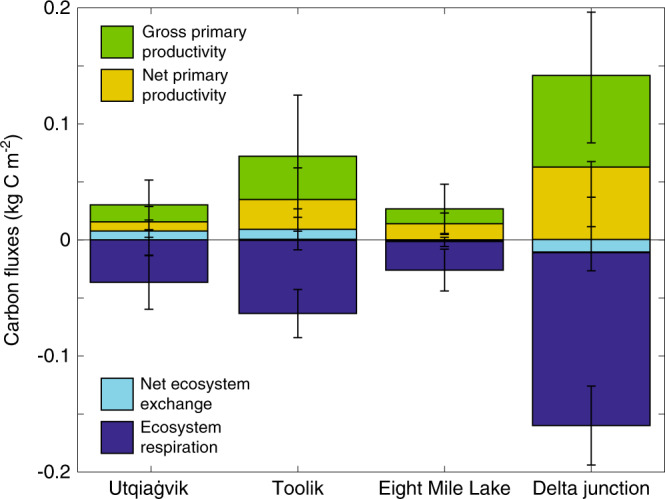
Modeled plant productivity is generally smaller than elevated ecosystem respiration (autotrophic + heterotrophic respiration) under short-term warming. Short-term changes in gross primary productivity (GPP), net primary productivity (NPP), net ecosystem exchange (NEE), and ecosystem respiration (R_eco_) under acute warming. The figure represents the mean of the different responses over 10 years (±standard deviation).

Modeled gross and net primary productivity under short-term warming showed a small statistically insignificant increase (Fig. 4), partly due to significant interannual variability. These vegetation responses are broadly consistent with conclusions from experimental warming studies at tundra sites^31^, observational data-syntheses^13,32^, and long-term monitoring programs^33^, which have demonstrated increased height and cover of deciduous shrubs and graminoids, and decreased mosses and lichen cover. However, while a decline in mosses and lichen is consistent across warming studies, evergreen shrubs have also been shown to respond positively to warming^34,35^, which was not modeled in these short-term simulations.

Clear site-level differences in the response to short-term warming was also modeled. Vegetation carbon and biomass responded more to warming in the warmer southern site, Delta Junction, relative to the northern, and cooler sites. A recent data-synthesis provides partial support for this finding. Elmendorf et al.^10^ found heterogeneity in plant responses to warming, with shrubs increasing with experimental warming in sites with higher mean annual temperatures and graminoids increasing under warming in cooler sites. In our short-term simulations, graminoid abundance was modeled to have a small positive response to warming at Delta Junction, Toolik, and Eight Mile Lake, consistent with warming experiments at this site^36^, with no change at Utqiaġvik.

### Multi-decadal perturbation

Replacing the stepwise experimental framework with longer-term experiments illustrated extensive differences in how the sites respond to perturbation (Fig. 2b). At Toolik and Delta Junction, ecosystem respiration outweighed the aboveground response (Fig. 2b) and sustained the trends predicted by short-term warming that Arctic ecosystems will become positive feedbacks to climate change, but with lower emergent temperature sensitivities (Fig. 3). Conversely, at Utqiaġvik and Eight Mile Lake, the activity of belowground communities was offset by interactions between vegetation and changes in landscape processes, resulting in a carbon sink over the long-term (Fig. 2b and Supplementary Fig. 1). We applied a process network approach^37^ to analyze interactions between variables and found that the most significant factors explaining whole-column SOC trends over the long-term were changes in plant productivity and physical state (soil temperature, soil moisture, ALD, and snowpack depth, Supplementary Fig. 1).

### Broad ecosystem responses

**Fig. 5:**
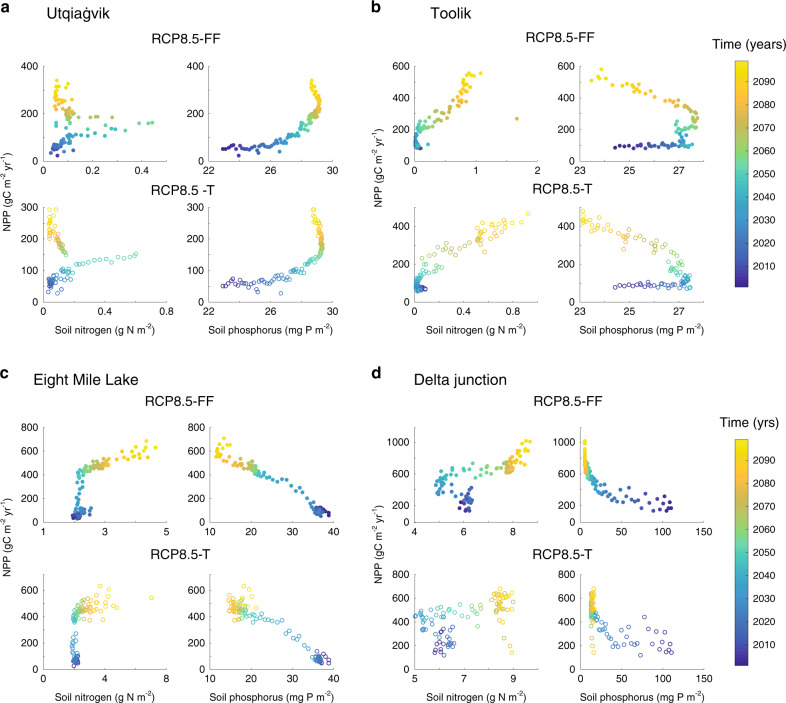
Nitrogen limitation constrains net primary productivity at most sites, but phosphorus appears to constrain net primary productivity at Utqiaġvik. Net primary productivity (calculated as an annual mean value in g C m^−2^ yr^−1^) plotted against soil availability of nitrogen (NH_4_^+^ + NO_3_^−^ in g N m^−2^) and phosphorus (g P m^−2^) at **a** Utqiaġvik, **b** Toolik, **c** Eight Mile Lake, and **d** Delta Junction. Phosphorus represents the modeled forms of SOM-P, labile-PO_4_^3−^, and mineral-associated PO_4_^3−^ available for plant and microbial uptake. NPP generally shows a positive relationship with soil nitrogen. However, a more complex relationship with phosphorus (a higher uptake of P towards the end of the century), suggests the possibility of emergent P-limitation over time. Plots show RCP8.5-FF (filled circles) and RCP8.5-T (unfilled circles). The plots are color-coded by time (right legend).

Under RCP8.5-FF and RCP8.5-T, air and soil temperatures and soil water content increased (Supplementary Table 4). Increasing moisture can increase thermal conductance, which can result in deepening of the active layer. These physical changes affected nutrient cycling, increasing nitrogen and phosphorus availability over time (Fig. 5). Changing nutrient availability can effect vegetation growth via two main depth-dependent processes: within shallower soil depths (~top 0.1 m), microbial mineralization rates increased as soil temperatures increased, accelerating nutrient cycling rates and liberating previously limiting nitrogen and phosphorus^38^ for uptake by plants with shallower rooting depths^39^. Deeper in the soil, thawing permafrost can release previously frozen inorganic nitrogen and phosphorus and enhance microbial decomposition^16^ of newly accessible organic matter^40^. Over time this accelerating nutrient cycling plays a significant role in determining the competitive dynamics of vegetation to warming.

In addition to increasing nutrient availability as ALD deepened, air temperature and soil moisture were the primary drivers behind the modeled 21st century site-average rise in NPP (~260 ± 150%), soil ecosystem respiration (autotrophic + heterotrophic, 280 ± 130%; Supplementary Fig. 2), and soil carbon (Fig. 2b and Supplementary Fig. 1). However, despite increasing nutrient availability, inorganic nitrogen availability still appeared to constrain NPP, as shown by a positive relationship between NPP and nitrogen availability at most sites (Fig. 5). This constraint could reflect increasing immobilization of nitrogen within microbial biomass^41^. Interestingly, Utqiaġvik showed a positive relationship between soil phosphorus concentration and NPP, indicative of potential phosphorus co-limitation of productivity in this region (Fig. 5). Previous studies support this assertion by showing, (1) a phosphorus control on productivity and carbon sequestration under sufficient nitrogen availability in tundra systems^42^ and (2) evidence for phosphorus control on ecosystem processes at Utqiaġvik^43^.

Over the 21st century, the modeled growing season lengthened, which contributed to increased plant productivity (Supplementary Fig. 2) and shrub biomass, the latter being consistent with recent observational^44^ and modeling^14^ analyses. It should be noted that while most modeling studies simulate prolonged seasonal vegetation activity, experiments and observations present a more complex picture whereby snow-free periods might increase, but the response in productivity is diverse^45^, and include evidence for increased^33^ and decreased^46^ vegetation productivity, abundance, biomass, or height. Furthermore, in instances where changes in aboveground biomass are not observed, significant changes can occur in belowground biomass^47^, which can decouple critical processes of the carbon cycle (i.e., productivity and respiration).

### Tundra vegetation responses to perturbation

At the two northern, colder, and nutrient poor sites (Utqiaġvik and Toolik), shrubs dominated the vegetation by 2100: at Utqiaġvik, evergreen and deciduous shrubs dominated the vegetation, representing ~70% of NPP, while at Toolik the deciduous shrubs account for ~60% of NPP, outcompeting evergreen shrubs for nutrients, and non-vascular plants for light via shading. Evergreen shrubs have lower nutrient demand, slower nutrient uptake, and greater nitrogen and phosphorus retention within leaves, shoots, and roots^48^. By contrast, the high rates of nutrient uptake and photosynthesis associated with deciduous shrubs result in a relatively rapid increase by 2100 in deciduous cover at Toolik as air temperatures warmed and nutrient availability increased^49^. The higher relative coverage of deciduous shrubs at Toolik relative to Utqiaġvik could partially explain the higher SOC loss at Toolik, since deciduous shrubs produce higher quality, more decomposable litter than evergreen shrubs.

At both Utqiaġvik and Toolik, graminoids generally maintained similar biomass until 2070, after which it slowly increased. The graminoids are modeled to have deeper roots than shrubs, and can therefore access nutrient mineralization occurring at the permafrost boundary. However, the negative effect of increased shading by the shrub dominated community offset the positive effects of increased nutrient availability.

These modeled shifts in vegetation are governed by plant functional type (PFT) traits related to thermal niche, carbon allocation, hydraulics, and growth strategies. In contrast to short-term manipulations, long-term simulations showed that plant responses to perturbation strongly affected SOC stocks at all sites (Supplementary Fig. 1). Eight Mile Lake, Toolik, and Utqiaġvik were modeled to have non-monotonic C cycle responses to perturbations over time. This pattern was most noticeable at Eight Mile Lake, which, although a strong SOC sink by 2100, peaked in mid-century and declined thereafter (by ~33% under the RCP8.5 scenarios; Fig. 2b). Therefore, equilibrium between SOC inputs and losses was not established over the 21st century. At this site, NPP (Supplementary Fig. 2) and vegetation biomass continue to increase over the century. However, as nutrient availability increased across the soil profile, belowground carbon allocation declined under both RCP8.5-FF (by 50%) and RCP8.5-T (by 80%, Supplementary Fig. 3).

The exudation of photosynthate belowground is tightly coupled to water and nutrient acquisition^50^, and plays an important role in the carbon cycle^51^. The proportion of belowground exudation falls within the range previously estimated for tundra plants^52^, while previous studies measuring belowground exudation within tundra ecosystems have observed either a decline^53^, or no change^54^ under warming. This is generally consistent with the current output described here (Supplementary Fig. 3). However, exudation is largely dependent on soil fertility, and nitrogen limitation can promote higher exudation^55^. In addition, plant species composition plays a large role in determining belowground exudation, as graminoids allocate more carbon belowground^56^. Therefore, the modeled drop in belowground exudation at Eight Mile Lake might be explained by increasing nutrient availability and shrub abundance, with lower nutrient requirements. The modeled decline in plant exudation did not occur at either Utqiaġvik or Toolik, which further supports the idea of strong nutrient limitation within these colder systems. However, the percentage of NPP allocated to belowground exudation does decrease over the 21st century (Supplementary Fig. 3). Uncertainty in the perturbation response of exudation, and its importance in the carbon cycle, highlights this area as a first-order research priority.

### Tundra soil carbon responses to perturbation

**Fig. 6:**
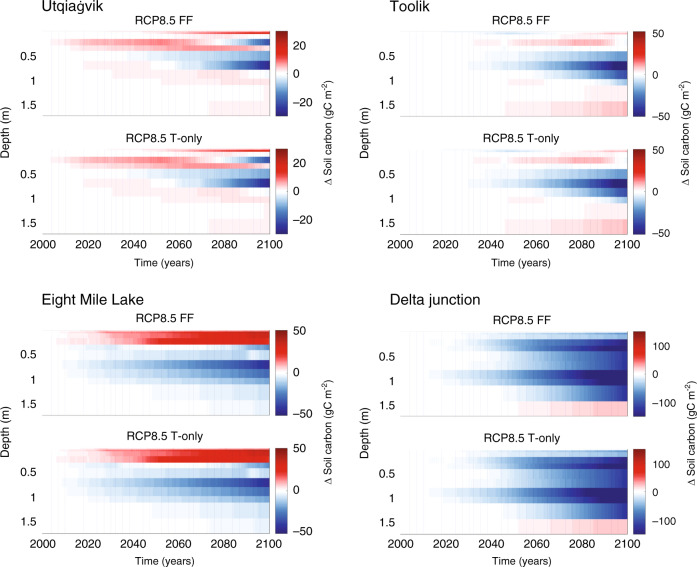
Soil carbon changes over time and depth. The panels show the depth-dependent changes in soil carbon (gC m^−2^) over time at each site. Red represents inputs of SOC, while blue indicates depths where soil organic carbon is lost. Note, the color-bar axes denoting changes in soil carbon differs from site to site.

Increasing vegetation productivity, aboveground biomass, and litterfall resulted in SOC accumulation within surface soils (<0.3 m) at Eight Mile Lake. However, SOC lost to heterotrophic respiration, increases over time, particularly towards the end of the century between 0.5 and 1 m (Fig. 6). These deeper hotspots of SOC turnover can occur for several reasons, including the increased availability of labile organic matter as the permafrost boundary deepens and an overall increasing rate of mineralization as kinetic energy limitations are alleviated by rising soil temperatures^57^. Furthermore, the contribution to SOC turnover attributable to wintertime heterotrophic respiration increases over time as soils warm, consistent with observations^58^. These hotspots of subsurface activity can partially offset carbon inputs by vegetation, emphasizing the importance of understanding the vulnerability of deep carbon stocks to warming^59^. Support for the long-term trajectory of SOC at Eight Mile Lake can also be found from the short-term warming manipulations at this site, which have shown both increased microbial activity^18^, and increasing plant productivity^60^.

Several of the mechanisms accounting for the sustained SOC sink at Eight Mile Lake apply to Utqiaġvik. In both cases, increased air temperature and nutrient availability increased plant productivity. However, Utqiaġvik is a much colder site than Eight Mile Lake (Supplementary Table 3). By 2100 the mean annual temperature at Utqiaġvik is ~1 °C (±3.3 °C standard deviation) and microbial decomposition remains energy limited^38^, with strong nutrient immobilization^61^. Nevertheless, gains in aboveground productivity are not entirely offset by those in belowground respiration (Supplementary Fig. 2) allowing for the small SOC accumulation relative to the baseline rates (Fig. 2). Therefore, dominant shrubs and graminoids become more competitive for nutrients over time. One potential reason for these changing ecosystem dynamics could be the deepening of the snowpack between 2000 and 2050 (Supplementary Table 3). Several snow fence field experiments have shown excess snow thermally insulates the soil. Thermal insulation over the winter can increase microbial mineralization and nutrient availability^62^. Plant uptake of this nitrogen can occur both under the snow and during the spring melt period^63^, particularly by shrubs^64^, leading to elevated foliar nitrogen content^60^. Nutrient uptake and storage can also occur after the growing season^65^, providing an advantage at the onset of the following growing season and spurring tundra vegetation growth.

The long-term SOC loss at Toolik represents an interesting counterpoint to the response at Utqiaġvik, and to EML. Toolik remains a source of carbon to the atmosphere throughout the 21st century, although the response to perturbation is relatively slow and the site is a carbon source under both short- and long-term warming. Under both perturbation scenarios this carbon trajectory occurs because Toolik, which is slightly warmer than Utqiagvik, showed a strong initial response of respiration to warming, and this response continues over time, particularly deeper in the soil profile at the permafrost boundary (Fig. 6). By contrast, the vegetation response appears to be the primary factor differentiating EML and Toolik. A deeper active layer, warmer air temperatures (Supplementary Table 3), and higher nutrient availability (Fig. 5) at EML promote more rapid plant growth and surface soil carbon accumulation (Fig. 6) over the century, relative to the Toolik site. Cooler temperatures, and lower nutrient availability at Toolik favors microbial immobilization of nutrients relative to the more rapid nutrient cycling at EML. This immobilization stimulates heterotrophic respiration under warming, mineralizing soil carbon. Over time, deciduous shrub biomass increases at the Toolik site, and higher C:N ratios and longer turnover times of woody biomass begins to offset these losses.

This balance between respiration and productivity stabilizes carbon losses in the latter half of the 21st Century, and Toolik begins to build up soil carbon, approaching, but not reaching, neutrality by 2100. One of the longest running warming experiments in the tundra, 20 years of warming at Toolik^8^, observed no net change in carbon stocks. However, the authors observed initial summertime warming over the first few years that diminished over time and concluded that the declining summertime soil respiration, coupled with greater litter inputs, may have offset any warming-driven carbon loses that likely occurred over those first few years. These differences between the experimentally manipulated warming and the imposed model warming partly explain discrepancies between observed and modeled soil carbon stock responses.

### Boreal Forest responses to perturbation

South of the tundra at the Delta Junction boreal site, losses from ecosystem respiration generally outweighed gains from increased productivity, leading to SOC losses across most of the soil column over the 21st century (Fig. 6). These SOC losses occurred concurrently with a slight increase in deciduous biomass (modeled as Aspen, and characterized by fast, inefficient growth rates and low nutrient efficiencies), within the Spruce-dominated forest, and a drop in the percent of photosynthate allocated belowground as exudate (~70% decrease by 2100). At Delta Junction, ecosystem respiration, vegetation productivity (e.g., GPP, NPP), and soil temperature changes were among the more important predictors of SOC dynamics (Supplementary Fig. 1). The strong carbon source at this site might be surprising given an increasing dominance by woody biomass. However, uncertainty remains as to how increased deciduous plant abundance might affect the carbon cycle. For example, an increase in slowly decomposing woody biomass could increase ecosystem carbon storage^66^. Alternatively, the high nitrogen content of deciduous litter might accelerate nitrogen cycling, and prime the decomposition of SOC^67^, although see^68^. Notably, the decomposition of deciduous litter was recently observed to occur more quickly within forested environments than within tundra^69^. Finally, this site showed the largest increase in wintertime respiration, increasing nearly six-fold by 2100, which can significantly contribute to SOC turnover and loss^58^.

A recent analysis by Cahoon et al.^70^ demonstrated temperature to be a strong determinant of net CO_2_ fluxes within high-latitude ecosystems. Warmer locations tended to be CO_2_ sources to the atmosphere regardless of vegetation cover, concluding that ecosystems increasingly dominated by shrubs accelerate carbon cycling, which could lead to carbon loss^70^. This carbon loss can be exacerbated by an increasing production of deciduous litter, which, with a lower C:N ratio, can be rapidly decomposed relative to other PFTs^69^. Finally, expanding boreal forests and increasing snowpack cover could affect climate through declines in surface albedo^71^, which can feedback positively to radiative forcing, and offset any gains in soil carbon^71^. However, we emphasized the need for longer-term (>10 year) data sets focused on the response of Boreal regions to climate change.

### Future research priorities

We propose several activities that could help bridge the gaps between short- and long-term responses and improve carbon cycle predictions. First, greater empirical understanding of belowground traits and phenology^51^, and particularly of the mechanistic controls on belowground plant allocation, the fate of exudates, and importance to the tundra carbon cycle is required. This knowledge is emerging within lower latitude systems^50^, but more work is needed in tundra and Boreal systems. Furthermore, integration of new understanding in models is required to correctly represent carbon cycle impacts from uncoupling above- and belowground phenology^47^.

Second, an improved representation of biogeochemical and biophysical processes involved in SOC stabilization or turnover is required. The longest running tundra warming study^8^ highlights mechanisms that have yet to be fully represented in models, including the importance of multi-trophic interactions, cryoturbation, and soil aggregation. Improved representation of microbial complexity (i.e., bacteria, archaea, and fungi), and their response to long-term perturbation (including shifting plant communities), is also critical for predicting the trajectory of soil carbon. How microbial guilds and competition and plant-litter composition determine carbon pool structure and size with increasing active layer depth remain critical unknowns. Moreover, further quantifying, at a variety of scales, the importance and trajectory of winter respiration remains a high priority^7,58,60^.

Third, attempts to understand the response of the Arctic carbon cycle to climate change have focused on experimental manipulations of air temperature^5,6^. While our simulations support the supposition that temperature is a major limiter on high-latitude ecosystems, additional drivers of ecosystem change, including precipitation and elevated CO_2_, affect the terrestrial carbon cycle. Our brief analysis of these individual forcing effects on ecosystem C cycling indicated that while temperature was a dominant driver of high-latitude ecosystem responses, elevated CO_2_ or precipitation changed the trajectory of SOC stocks over time (Supplementary Fig. 4). While long-term data sets characterizing changes in vegetation over decadal-time scales are valuable for benchmarking model responses under fully forced scenarios, additional data is required to parse out the contribution of different drivers (e.g., CO_2_, precipitation). Therefore, further field experiments focusing on how individual and combined non-temperature based drivers of ecosystem function affect high-latitude ecosystems would contribute to improved prediction of the carbon cycle under climate change.

The trajectory of high-latitude soil carbon under climate change remains a source of uncertainty^72^. Short-term warming stimulates microbial decomposition, in line with observations from experimental manipulations and monitoring^18^. Here, we show that mechanisms emerging over century-long climate change simulations moderate soil carbon losses leading to a lower carbon feedback to climate than would be predicted from the emergent temperature sensitivities derived from short-term warming experiments. Across the 21st Century, elevated plant productivity, in response to thermal, hydrological, and nutrient transformations belowground, offsets soil carbon loss through microbial decomposition, for which there is support within the contemporary observational literature^8^. Furthermore, these long-term simulations illustrate clear regional heterogeneity, which can be attributed to subtle changes in vegetation and soil thermal properties. This heterogeneity is a feature of high-latitude ecosystems^10,72^ and characterizing the importance of this heterogeneity with respect to the carbon cycle remains a high research priority.

## Methods

### *Ecosys* model

The interdependent physical, hydrological, and biological processes governing the ecosystem response to warming are simulated by the mathematical model, *Ecosys*. Appendices describing the model equation have been published previously^24,28^. In brief*, Ecosys* is an hourly time-step land model with multiple canopy and soil layers and fully coupled carbon, energy, water, and nutrient cycles solved at an hourly time step^7^. Surface energy and water exchanges drive soil heat and water transfers to determine soil temperatures and water contents^8^. These transfers drive soil freezing and thawing and, hence, active layer depth, through the general heat flux equation. Carbon uptake is controlled by plant water status calculated from convergence solutions that equilibrate total root water uptake with transpiration^9^. Atmospheric warming increases surface heat advection, soil heat transfers, and hence active layer depth.

*Ecosys* represents fully coupled plant-microbe-soil carbon and nutrient cycling driven by the energetics and kinetics of aerobic and anaerobic oxidation-reduction reactions, fully prognostic dynamics of permafrost and its effects on active layer hydrology driven by basic processes for transfer and transformation of energy and water, and acclimation of all biological processes to warming^10,11^. *Ecosys* represents multiple canopy and soil layers allowing for mechanistic plant functional type (PTF) competition for light and water. PFTs were constructed from published descriptions of the key vegetation types for each site^1–6^. A range of PFTs were represented in the simulations, including evergreen and deciduous trees and shrubs, lichen, heath, nitrogen-fixing moss, and C3-grasses. Competitive dynamics amongst Arctic PFTs are governed by distinct functional traits^12^. These traits generally regulate resource acquisition and allocation strategies that determine variability in phenology, irradiance, CO_2_ fixation rate, and water uptake among PFTs, and therefore, drive their ability to grow and compete.

There are several PFTs explicitly assigned in *Ecosys* (e.g., traits related to CO2 fixation, phenology, root traits, and mycorrhizal growth), but five basic traits (leaf mass area, leaf clumping, foliar nutrient content, foliar nutrient retention, and root hydraulic conductivity) are critical in determining the ecological strategy of an individual PFTs. A recent publication has highlighted in detail the significance of differences in these traits for vegetation dynamics in Arctic tundra under a changing climate^12^. PFTs also compete with distinct microbial guilds for common nutrient (nitrogen and phosphorus) pools held across multilayer soil profiles. PFT nutrient uptake is determined by root length and density and driven by allocation of non-structural carbon, nitrogen, and phosphorus, the rate of which is determined by concentration gradients in non-structural carbon, nitrogen, and phosphorus within each PFT arising from CO2 fixation or root uptake of nitrogen and phosphorus relative to consumption by autotrophic respiration. Multiple microbial guilds representing aerobic and anaerobic (e.g., denitrification, fermentation, methanogenesis, methanotrophy) heterotrophic and autotrophic (e.g., nitrification and methanotrophy) metabolisms are represented in *Ecosys*. The rate of organic matter decomposition and turnover is determined by the rate of heterotrophic respiration and constrained by microbial nutrient availability (i.e., C:N and C:P ratios), soil temperature, oxygen availability, and organic carbon concentration. All microbial populations seek to maintain stoichiometric balance through mineralization and immobilization of NH_4_^+^, NO_3_^−^, and H_2_PO_4_^−^. Uptake of these nutrients is determined by Michaelis–Menton kinetics, and constrained by surface area: volume ratio. Microbial biomass growth is dependent on the energy yield (∆G) gained during growth respiration. Growth respiration represents the excess total heterotrophic respiration after accounting for cellular maintenance respiration. In the event that maintenance respiration exceeds total heterotrophic respiration, microbial mortality occurs. Mortality also occurs under first-order decay rates. The key algorithms governing the modeling of ecological responses to warming in tundra soils have been described recently^13,14^, and the reader is referred to those manuscripts for specific details. The growth and activity of microbial guilds is not prescribed by season. Thermal insulation by deep a snowpack, which permit the persistence of aqueous conditions within soil macro-, or micropores permit, in turn, the activity of the different microbial guilds down to very low bulk soil temperatures (e.g., >−10 °C). It is therefore possible to simulate changes in wintertime respiration under RCP85 scenarios, consistent with recent findings^58^.

*Ecosys* has been applied to model the ecological controls on ecosystem processes and the whole ecosystem response to changing climate across different ecosystems, including within high-latitude soils; Arctic tundra^11,12^, and fens^13,14^, boreal forests^8,15–18^. We note that the *Ecosys* model performs extremely well in replicating contemporary flux tower estimates of carbon exchange in Alaskan ecosystems^19,20^, and is therefore an appropriate model for testing how Arctic ecosystems response to changing climate. The parameters and algorithms governing the modeling of the tundra ecosystem have been described in detail previously^21^.

The four sites, Utqiaġvik, Toolik, Eight Mile Lake, and Delta Junction were represented as individual column experiments. The atmospheric forcing data, providing inputs for air temperature, precipitation, downward shortwave radiation, relative humidity, and wind speed for each site, were taken from the North American Regional Reanalysis (NARR), a long-term weather dataset originally produced at the National Oceanic and Land Administration (NOAA) National Centers for Environmental Prediction (NCEP) Global Reanalysis^22^.

Where possible these model drivers were supplemented by site-specific data. For example, long-term studies at Toolik and Eight Mile Lake maintain sufficient data archives for environmental variables, including temperature and precipitation (see http://www.lter.uaf.edu/data/andhttps://toolik.alaska.edu/edc/), to allow substitution of the NARR variables within the model drivers. Similarly, the Next Generation Ecosystem Experiment-Arctic maintains a data archive for the Utqiaġvik site (http://ngee-arctic.ornl.gov/). At each site, soils were represented to a depth of 2 m. Soil properties were initialized with attributes from the Unified North America Soil Map (UNASM)^23^, however, site-specific values for edaphic factors and land cover were also used to initialize the model. Where possible bulk density, soil pH, sand, silt, and clay content were compiled from published observations. Soil organic carbon was initialized with the Northern Cirumpolar Soil Carbon Database (NCSCD)^24^. Changes in soil ice content were used to calculate the soil thaw depth, and modeled using a heat flux equation driven by surface energy exchange and subsurface heat transfer (see Grant and Pattey^25^, for further details). These fluxes drive freeze-thaw dynamics and temperature in the snowpack, surface litter and soil layers^13^. *Ecosys* was benchmarked using a meta-analysis of carbon responses to warming. The meta-analysis was limited to the sites included in the simulations and to two response variables, Ecosystem Respiration (Reco) and GPP). The bulk of the current data had been prepared for the previous publication^28^, however, this database was updated here to include publications up to March, 2017. Additional data was collected using the matlab script GRABIT (available online, https://www.mathworks.com/matlabcentral/fileexchange/7173-Grabit).

### Model testing

Baseline *Ecosys* simulations were run from 1900 to 2000 under dynamic climate, atmospheric CO_2_ concentrations^73^, and nitrogen deposition^74^ that enabled the model to attain a spin up prior to the onset of perturbation. In order to test the response of the model to perturbation we replicated warming experiments conducted at the different sites (Table 1). To achieve this we reproduced the magnitude of warming over the same period the original experiments were run for (i.e., after five, three, five, and 2 years of warming at Utqiaġvik, Toolik, Eight Mile Lake, and Delta Junction, respectively^7,9,26,60,75^). It should be noted that, given the limited observations available, we chose (and parameterized) study sites that capture the general characteristics of their region. For example, the Delta Junction site is parameterized as a general boreal forest site, with relevant plant functional types (moss, lichen, graminoids, and evergreen and deciduous trees). Although clearly insufficient to make a pan-Arctic extrapolation, we believe this approach permits some generalizability from this study. At Utqiaġvik and Toolik OTC experiments were established during the growing season in 1995 and CO_2_ fluxes measured using a LI-6200 portable photosynthesis system (LI-COR, Lincoln, Nebraska, USA) in 2000 and 2001 (Utqiaġvik), and in 1997 and 1998 (Toolik). Measurements were conducted every 4 h over 24 h. Air temperatures within the OTC increased by 1–2 °C. Details on the calculation of gross ecosystem photosynthesis and ecosystem respiration can be found in the original text^26^.

**Table 1:** Data sets used in model testing.

Authors	Site	Study title	Time period for warming (sampling year)	Journal (year)	DOI	Funding agency
Oberbauer et al.^26^	Utqiaġvik	Tundra CO_2_ fluxes in response to experimental warming across latitudinal and moisture gradients	5 years (2001)	Ecological Monographs (2007)	10.1890/06-0649	National Science Foundation (USA)
	Toolik		3 years (1998)			
Mauritz et al.^7^	Eight Mile Lake	Non-linear CO_2_ flux response to 7 years of experimentally induced permafrost thaw	5 years (2015)	Global Change Biology (2017)	10.1111/gcb.13661	Department of Energy/ National Science Foundation (USA)
Allison & Treseder^9^	Delta Junction	Warming and drying suppress microbial activity and carbon cycling in boreal forest soils.	2 years (2007)	Global Change Biology (2008)	10.1111/j.1365-2486.2008.01716.x	Department of Energy/ NOAA/ National Science Foundation (USA)

The Eight Mile Lake experiment was initiated in 2008/ 2009^7,60^, and continued until 2015. Growing season warming was achieved through the placement of OTCs, increasing air temperatures increased by 0.4 °C. Net ecosystem exchange was monitored during the growing season using an automated chamber system connected to an infrared gas analyzer (LI-820, Li-COR, Lincoln, Nebraska, USA). Warming plots at Delta Junction were established in 2004, and warming experiments conducted between 2005 and 2007^9^. In contrast to the other studies, warming at Delta Junction was achieved using closed top chambers (greenhouses), which warmed soil temperatures ~0.5 °C. Soil respiration was monitored using an infrared gas analyzer (PP Systems EGM-4, Amesbury, MA, USA) every 1–2 months during the growing season.

The in situ ecosystem warming simulations were parameterized to reflect open-top chamber field experiments; the soil was warmed during the growing season by scaling the aerodynamic resistance, in accordance with a previously employed approach^27^ to match field observations of warming at each of the different sites while keeping sufficient spatial variability of the warming. We then compared the relative change in gross primary productivity (ΔGPP = GPP_warming_ − GPP_ambient_), ecosystem respiration (ΔR_eco_), and active layer depth (ΔALD) from the observations with the simulations. Significant differences between the empirical and simulated response to warming were analyzed using a two-way analysis of variance.

Finally, to compare the performance of the model across the spatially diverse sites we used a meta-analysis to visualize the divergence in GPP and R_eco_ under warming from the control treatment, following a previously published approach^27^. We note, however, that the site describing the software originally used for performing this meta-analysis (http://www.metawinsoft.com/) is no longer available. However, we note that similar, open-access software is available, notably OpenMEE (http://www.cebm.brown.edu/openmee), the meta package (https://github.com/guido-s/meta), and the metafor package (http://www.metafor-project.org).

For each variable (GPP or R_eco_) we combined measurements from the control and warming experiments across all four sites for (a) the empirical data (taken from refs. ^7,9,26,60,75^) and (b) the model outputs (i.e., the raw interannual data for the control and warming studies). A response metric was calculated as the natural log of treatment group (i.e., warming) relative to an ambient control:

2 $${\mathrm{lnR}} = {\mathrm{ln}}\left( {\frac{{\overline {\mathrm{X}} ^{\mathrm{T}}}}{{\overline {\mathrm{X}} ^{\mathrm{A}}}}} \right)$$

Where $\overline {\mathrm{X}} ^{\mathrm{T}}$ and $\overline {\mathrm{X}} ^{\mathrm{A}}$ are the mean values for the treatment and ambient response variable, respectively. The sampling variance (V_InR_) was calculated as:

3 $${\mathrm{V}}_{{\mathrm{lnR}}} = \frac{{\left( {{\mathrm{s}}^{\mathrm{T}}} \right)^2}}{{{\mathrm{N}}^{\mathrm{T}}\left( {\overline {\mathrm{X}} ^{\mathrm{T}}} \right)^2}} + \frac{{\left( {{\mathrm{s}}^{\mathrm{A}}} \right)^2}}{{{\mathrm{N}}^{\mathrm{A}}\left( {\overline {\mathrm{X}} ^{\mathrm{A}}} \right)^2}}$$

Where s^T^ and s^A^ represent the normalized standard deviations around the mean values and N^T^ and N^A^ are the number of replicate studies from the warming treatment and ambient controls, respectively. The effect size for different response metrics (e.g., GPP or R_eco_) was subsequently calculated using a weighted average value, where the weight for the i^th^ study is the reciprocal of its sampling variance. The GPP and R_eco_ effect sizes for the empirical data and the model simulations are then visualized using a forest plot, which shows the % divergence from the control. A one-way anova found no significant differences between the % divergence between the model output and the empirical data.

### Short-term warming simulations

The short-term experimental simulations were run by restarting the baseline model simulations described above at the year 2000 and running to 2010 under control (i.e., no warming) conditions and under a short-term warming scenario. Under these conditions soil temperature elevation ranged from 0.35 °C at Eight Mile Lake^7^ to 1.2 °C at Toolik^7,9,26,60,75^.

### Long-term simulations

Additional simulations examined the ecosystem response to long-term climate perturbation through two different experimental scenarios. The first scenario examines the ecosystem response to warming only, in line with the short-term experimental conditions. These simulations, termed warming, increase air temperature monotonically following the representative concentration pathway 8.5 (RCP8.5), obtained from ensemble projections, downscaled and averaged from 15 CMIP5 models. Climate forcing for the second experiment, termed long-term full forcing, also follows the RCP8.5 scenario, however, in addition to changing air temperature, also projects forcing of precipitation, solar radiation, atmospheric humidity, and atmospheric CO_2_ concentration. Two further simulations isolated the influence of single variables, changing precipitation and elevated CO2. RCP8.5 was followed for each variable, while maintaining all other forcing factors at 2010 values. Each experimental was forced with the different RCP8.5 scenarios for years 2000–2100, restarting from the baseline simulations, which represent the years 1900–2000. Baseline simulations were also run out to 2100 for comparison (Supplementary Figure 5).

### Causality modeling with information theory

We treat the complex aboveground and belowground interactions as a coupled process network^37^, in which directional impacts from one (e.g., plant carbon productivity) to the other (e.g., soil carbon budget) could be quantitatively inferred by Shannon information entropy (*H*) and its transfer (TE) (unit bits):

4 $$H = - \mathop {\sum}\limits_{i = 1}^n {p\left( {x_i} \right)\log _2p\left( {x_i} \right)} $$

5 $$T_{X - > Y} = \mathop {\sum}\limits_{y_i,y_{i - 1},x_{i - j}} {p\left( {y_i,y_{i - 1},x_{i - j}} \right)\log _2\frac{{p\left( {y_i\left| {y_{i - 1},x_{i - j}} \right.} \right)}}{{p\left( {y_i\left| {y_{i - 1}} \right.} \right)}}} $$

where *p(x)* is probability density function (PDF) of *x*, *p(y*_*i*_*,y*_*i-1*_*,x*_*i-j*_*)* is the joint PDF of current time step *y*_*i*_, previous time step of *y*_*i*_, and *j*^th^ time step before of *x*_*i*_. *p(y*_*i*_*|y*_*i−1*_*,x*_*i−j*_*)* and *p(y*_*i*_*|y*_*i−1*_*)* denote conditional PDF of the corresponding variables. For example, the information entropy transfer from plant photosynthesis processes (GPP) to soil heterotrophic respiration processes (RH) is then calculated as Shannon entropy reduction (uncertainty reduction) of present RH given the historical GPP records and also excluded the influence from previous time step RH. The significant threshold of *TE*_*GPP−>RH*_ is by first randomly shuffling GPP and RH time series, then calculate the shuffled *TE*_*GPP−>RH*_, assuming the randomly shuffle breaks the dependency between GPP and RH. We applied this causality modeling approach to *ecosys* simulated time series of system carbon, nitrogen, phosphorus, water, and heat fluxes as well as associated pools and status at Utqiaġvik, Toolik Lake, Eight Mile Lake, and Delta Junction.

## Supplementary information

Supplementary Information

## Data Availability

In accordance with US-DOE data policy, the data presented in this manuscript, and the matlab scripts used to generate the figures has been deposited to the ESS-DIVE repository (https://ess-dive.lbl.gov/) at 10.15485/1670465.

## Code availability

The *ecosys* source code is publically available at https://github.com/jinyun1tang/ECOSYS.

## References

[1] Friedlingstein P (2006). Climate–carbon cycle feedback analysis: results from the C ^4^ MIP model intercomparison. J. Clim..

[2] Hugelius G (2013). A new data set for estimating organic carbon storage to 3 m depth in soils of the northern circumpolar permafrost region. Earth Syst. Sci. Data.

[3] Hugelius G (2014). Estimated stocks of circumpolar permafrost carbon with quantified uncertainty ranges and identified data gaps. Biogeosciences.

[4] Serreze MC, Barry RG (2011). Processes and impacts of Arctic amplification: a research synthesis. Glob. Planet. Change.

[5] Schuur EAG (2015). Climate change and the permafrost carbon feedback. Nature.

[6] Elberling B (2013). Long-term CO2 production following permafrost thaw. Nat. Clim. Change.

[7] Mauritz M (2017). Nonlinear CO_2_ flux response to 7 years of experimentally induced permafrost thaw. Glob. Change Biol..

[8] Sistla SA (2013). Long-term warming restructures Arctic tundra without changing net soil carbon storage. Nature.

[9] Allison SD, Treseder KK (2008). Warming and drying suppress microbial activity and carbon cycling in boreal forest soils. Glob. Change Biol..

[10] Elmendorf SC (2012). Global assessment of experimental climate warming on tundra vegetation: heterogeneity over space and time: warming effects on tundra vegetation. Ecol. Lett..

[11] Bokhorst S (2013). Variable temperature effects of Open Top Chambers at polar and alpine sites explained by irradiance and snow depth. Glob. Change Biol..

[12] Arft AM (1999). Responses of tundra plants to experimental warming: meta-analysis of the international tundra experiment. Ecol. Monogr..

[13] Elmendorf SC (2012). Plot-scale evidence of tundra vegetation change and links to recent summer warming. Nat. Clim. Change.

[14] Mekonnen ZA, Riley WJ, Grant RF (2018). Accelerated nutrient cycling and increased light competition will lead to 21st century shrub expansion in North American Arctic Tundra. J. Geophys. Res. Biogeosci..

[15] Elmendorf SC (2015). Experiment, monitoring, and gradient methods used to infer climate change effects on plant communities yield consistent patterns. Proc. Natl Acad. Sci. USA.

[16] Rustad L (2001). A meta-analysis of the response of soil respiration, net nitrogen mineralization, and aboveground plant growth to experimental ecosystem warming. Oecologia.

[17] Zhu Z (2016). Greening of the Earth and its drivers. Nat. Clim. Change.

[18] Xue K (2016). Tundra soil carbon is vulnerable to rapid microbial decomposition under climate warming. Nat. Clim. Change.

[19] Kwon MJ (2019). Drainage enhances modern soil carbon contribution but reduces old soil carbon contribution to ecosystem respiration in tundra ecosystems. Glob. Change Biol..

[20] Reich PB, Hobbie SE, Lee TD, Pastore MA (2018). Unexpected reversal of C_3_ versus C_4_ grass response to elevated CO_2_ during a 20-year field experiment. Science.

[21] Melillo JM (2017). Long-term pattern and magnitude of soil carbon feedback to the climate system in a warming world. Science.

[22] Andresen LC (2016). Shifting impacts of climate change. Adv. Ecol. Res..

[23] Lawrence DM, Slater AG, Romanovsky VE, Nicolsky DJ (2008). Sensitivity of a model projection of near-surface permafrost degradation to soil column depth and representation of soil organic matter. J. Geophys. Res..

[24] Grant RF (2017). Mathematical modelling of arctic polygonal tundra with *Ecosys*: 1. Microtopography determines how active layer depths respond to changes in temperature and precipitation: active layer depth in polygonal tundra. J. Geophys. Res. Biogeosci..

[25] Yamori W, Hikosaka K, Way DA (2014). Temperature response of photosynthesis in C3, C4, and CAM plants: temperature acclimation and temperature adaptation. Photosynth. Res..

[26] Oberbauer SF (2007). Tundra CO_2_ fluxes in response to experimental warming across latitudinal and moisture gradients. Ecol. Monogr..

[27] Bouskill NJ, Riley WJ, Tang JY (2014). Meta-analysis of high-latitude nitrogen-addition and warming studies implies ecological mechanisms overlooked by land models. Biogeosciences.

[28] Grant RF, Humphreys ER, Lafleur PM (2015). Ecosystem CO_2_ and CH_4_ exchange in a mixed tundra and a fen within a hydrologically diverse Arctic landscape: 1. Modeling versus measurements: CO_2_ and CH_4_ exchange in the Arctic. J. Geophys. Res. Biogeosci..

[29] Monson RK (2006). Winter forest soil respiration controlled by climate and microbial community composition. Nature.

[30] Deane-Coe KK (2015). Experimental warming alters productivity and isotopic signatures of tundra mosses. Ecosystems.

[31] Walker MD (2006). Plant community responses to experimental warming across the tundra biome. Proc. Natl Acad. Sci. USA.

[32] Bjorkman AD (2018). Plant functional trait change across a warming tundra biome. Nature.

[33] Myers-Smith IH (2019). Eighteen years of ecological monitoring reveals multiple lines of evidence for tundra vegetation change. Ecol. Monogr..

[34] Hudson JMG, Henry GHR (2009). Increased plant biomass in a High Arctic heath community from 1981 to 2008. Ecology.

[35] Weijers S (2012). No divergence in Cassiope tetragona: persistence of growth response along a latitudinal temperature gradient and under multi-year experimental warming. Ann. Bot..

[36] Hicks Pries CE, Schuur EAG, Natali SM, Crummer KG (2016). Old soil carbon losses increase with ecosystem respiration in experimentally thawed tundra. Nat. Clim. Change.

[37] Ruddell BL, Kumar P (2009). Ecohydrologic process networks: 2. Analysis and characterization: Ecohydrologic Process Networks, 2.. Water Resour. Res.

[38] Nadelhoffer KJ, Giblin AE, Shaver GR, Laundre JA (1991). Effects of temperature and substrate quality on element mineralization in six arctic soils. Ecology.

[39] Wang P (2017). Above- and below-ground responses of four tundra plant functional types to deep soil heating and surface soil fertilization. J. Ecol..

[40] Monteux S (2018). Long-term in situ permafrost thaw effects on bacterial communities and potential aerobic respiration. ISME J..

[41] Booth MS, Stark JM, Rastetter E (2005). Controls on nitrogen cycling in terrestrial ecosystems: A synthetic analysis of literature data. Ecol. Monogr..

[42] Street LE, Mielke N, Woodin SJ (2018). Phosphorus availability determines the response of tundra ecosystem carbon stocks to nitrogen enrichment. Ecosystems.

[43] Chapin FS, Barsdate RJ, Barèl D, Barel D (1978). Phosphorus cycling in alaskan coastal tundra: a hypothesis for the regulation of nutrient cycling. Oikos.

[44] Myers-Smith IH (2011). Shrub expansion in tundra ecosystems: dynamics, impacts and research priorities. Environ. Res. Lett..

[45] Myers-Smith, I. et al. *Complexity revealed in the greening of the Arctic*. 10.32942/osf.io/mzyjk (2019).

[46] Bhatt U (2013). Recent declines in warming and vegetation greening trends over pan-arctic tundra. Remote Sens.

[47] Blume-Werry G, Wilson SD, Kreyling J, Milbau A (2016). The hidden season: growing season is 50% longer below than above ground along an arctic elevation gradient. New Phytol..

[48] Freschet GT, Cornelissen JHC, van Logtestijn RSP, Aerts R (2010). Evidence of the ‘plant economics spectrum’ in a subarctic flora. J. Ecol..

[49] Chapin FS, Shaver GR, Giblin AE, Nadelhoffer KJ, Laundre JA (1995). Responses of arctic tundra to experimental and observed changes in climate. Ecology.

[50] Canarini A, Kaiser C, Merchant A, Richter A, Wanek W (2019). Root exudation of primary metabolites: mechanisms and their roles in plant responses to environmental stimuli. Front. Plant Sci..

[51] Iversen CM (2015). The unseen iceberg: plant roots in arctic tundra. New Phytol..

[52] Loya WM (2002). Pulse-labeling studies of carbon cycling in arctic tundra ecosystems: Contribution of photosynthates to soil organic matter: photosynthate contribution to soil C storage. Glob. Biogeochem. Cycles.

[53] DeMarco J, Mack MC, Bret-Harte MS, Burton M, Shaver GR (2014). Long-term experimental warming and nutrient additions increase productivity in tall deciduous shrub tundra. Ecosphere.

[54] Wang P (2016). Belowground plant biomass allocation in tundra ecosystems and its relationship with temperature. Environ. Res. Lett..

[55] Yin H (2013). Enhanced root exudation stimulates soil nitrogen transformations in a subalpine coniferous forest under experimental warming. Glob. Change Biol..

[56] Ström L, Tagesson T, Mastepanov M, Christensen TR (2012). Presence of Eriophorum scheuchzeri enhances substrate availability and methane emission in an Arctic wetland. Soil Biol. Biochem..

[57] Mackelprang R (2011). Metagenomic analysis of a permafrost microbial community reveals a rapid response to thaw. Nature.

[58] Natali SM (2019). Large loss of CO_2_ in winter observed across the northern permafrost region. Nat. Clim. Change.

[59] Hicks Pries CE, Castanha C, Porras RC, Torn MS (2017). The whole-soil carbon flux in response to warming. Science.

[60] Natali SM, Schuur EAG, Rubin RL (2012). Increased plant productivity in Alaskan tundra as a result of experimental warming of soil and permafrost: Increased plant productivity in Alaskan tundra. J. Ecol..

[61] Weintraub MN, Schimel JP (2003). Interactions between carbon and nitrogen mineralization and soil organic matter chemistry in Arctic tundra soils. Ecosystems.

[62] Schimel JP, Bilbrough C, Welker JM (2004). Increased snow depth affects microbial activity and nitrogen mineralization in two Arctic tundra communities. Soil Biol. Biochem..

[63] Bilbrough CJ, Welker JM, Bowman WD (2000). Early spring nitrogen uptake by snow-covered plants: a comparison of arctic and alpine plant function under the snowpack. Arct. Antarct. Alp. Res..

[64] Weintraub MN, Schimel JP (2005). The seasonal dynamics of amino acids and other nutrients in Alaskan Arctic tundra soils. Biogeochemistry.

[65] Riley WJ, Zhu Q, Tang JY (2018). Weaker land–climate feedbacks from nutrient uptake during photosynthesis-inactive periods. Nat. Clim. Change.

[66] Mekonnen ZA, Riley WJ, Grant RF (2018). 21st century tundra shrubification could enhance net carbon uptake of North America Arctic tundra under an RCP8.5 climate trajectory. Environ. Res. Lett..

[67] Weintraub MN, Schimel JP (2005). Nitrogen cycling and the spread of shrubs control changes in the carbon balance of arctic tundra ecosystems. BioScience.

[68] Cornelissen JHC (2007). Global negative vegetation feedback to climate warming responses of leaf litter decomposition rates in cold biomes. Ecol. Lett..

[69] Parker TC (2018). Exploring drivers of litter decomposition in a greening Arctic: results from a transplant experiment across a treeline. Ecology.

[70] Cahoon SMP, Sullivan PF, Shaver GR, Welker JM, Post E (2012). Interactions among shrub cover and the soil microclimate may determine future Arctic carbon budgets. Ecol. Lett..

[71] Betts RA (2000). Offset of the potential carbon sink from boreal forestation by decreases in surface albedo. Nature.

[72] Fisher JB (2014). Carbon cycle uncertainty in the Alaskan Arctic. Biogeosciences.

[73] Meinshausen M (2011). The RCP greenhouse gas concentrations and their extensions from 1765 to 2300. Clim. Change.

[74] Dentener F (2006). Nitrogen and sulfur deposition on regional and global scales: a multimodel evaluation. Glob. Biogeochem. Cycles.

[75] Leffler AJ, Klein ES, Oberbauer SF, Welker JM (2016). Coupled long-term summer warming and deeper snow alters species composition and stimulates gross primary productivity in tussock tundra. Oecologia.

