# Supplementary material for: Alaskan carbon-climate feedbacks will be weaker than inferred from short-term experiments
Source: Nat Commun. 2020 Nov 16;11:5798. doi: 10.1038/s41467-020-19574-3 (PMC7670472; doi:10.1038/s41467-020-19574-3)
Supplement: Supplementary file 1 — Supplementary Information [file 41467_2020_19574_MOESM1_ESM.pdf]

Alaskan carbon-climate feedbacks will be weaker than inferred from short-term manipulations

Nicholas J Bouskill[1], William J Riley[1], Qing Zhu[1], Zelalem Mekonnen[1], Robert Grant[2].

[1]Climate and Ecosystem Sciences Division, Lawrence Berkerley National Laboratory, Berkeley, CA, 94720.

[2]Department of Renewable Resources, University of Alberta, Edmonton, Canada.

Supplementary Notes

Parsing out forcing factors: Attempts to understand the response of the Arctic carbon cycle to climate change have focused strongly on experimental manipulations of air temperature[1–3]. We note here that the predicted SOC stock differences between the warming and fully forced scenarios were small, indicating temperature is a strong controlling factor in high latitude ecosystems. However, additional drivers of ecosystem change, including changing precipitation[4,5] and elevated CO_2 (eCO_2)[6] affect plant and microbial activity and therefore the terrestrial carbon cycle. Therefore, to more fully characterize the factors driving the changes in SOC (as described above) we performed two additional 100-year simulations (2000 – 2100) at each site aimed at isolating the affect of increasing precipitation and elevated atmospheric CO_2.

At Utqiaġvik and Toolik RCP8.5-ppt and RCP8.5-CO_2 largely replicated the previously described trajectories of SOM under RCP8.5-FF and RCP8.5-T. For example, increasing atmospheric CO_2 resulted in a stronger carbon sink at Utqiaġvik, and a slightly weaker carbon source at Toolik. By contrast, comparison between the different RCP8.5 scenarios at Eight Mile Lake and Delta Junction demonstrate that increasing temperature explains a significant amount of the change in SOC over time. Despite difference in the sign of the response at these two sites, increasing just precipitation or atmospheric CO_2 resulted in weaker feedbacks on SOC than when considering just temperature or fully forced simulations. While

this reinforces the view that temperature is a primary driver of soil carbon fate and plant phenology[7] at high latitudes, it also argues for a greater understanding of the relationship between these individual drivers and ecosystem responses.

Contextualizing this output is challenging due to the small number of field studies manipulating precipitation or CO_2 concentrations in high latitude ecosystems. A number of studies have examined the hypothesis that water limitation constrains productivity of tundra vegetation. Most studies find either no influence of increased summer precipitation on plant productivity[8,9] or a small positive effect[10]. However, even this small number of manipulation studies have exhibited clear spatial heterogeneity in response[10]. Similarly, there are also far fewer high-latitude CO_2 enrichment experiments than there have been in temperate systems[11]. Experiments over the course of a growing season have shown the response to elevated CO_2 to be related to the availability of nutrients and light in both tundra[12] and boreal[13] systems.

While temperature is clearly an important driver of high-latitude ecosystem response there is not currently enough data to draw strong conclusions on the role of additional forcing factors, such as precipitation or CO_2 over longer time scales or across the significant spatial heterogeneity of tundra systems. The feedback between atmospheric CO_2 and soil carbon content clearly warrants further study within high-latitude systems. Therefore, we recommend further studies that attempt to understand how additional drivers of ecosystem function perturb high-latitude ecosystems over long time frames.

Supplementary Tables

Supplementary Table 1. Seasonal increases in average maximum and minimum temperatures and increases in precipitation and atmospheric CO_2 concentration (C_a) relative to current values from 1981-2010 to 2071 – 2100 under a RCP 8.5 emission scenario downscaled and averaged across 15 CMIP5 models for the grid cell in which BEO is located.

	**Winter (DJF)**	**Spring (MAM)**	**Summer (JJA)**	**Autumn SON)**
Max. Temp. (ºC)	10.97	7.08	4.53	7.25
Min. Temp. (ºC)	12.80	8.28	4.84	8.30
Precipitation (-)	1.34	1.52	1.28	1.34
C_a (-)	2.37	2.37	2.37	2.37

Supplementary Table 2. Simulated site characteristics under short-term warming. The table shows the change in a given value relative to the baseline values (most of the baseline values are provided in table S4). Pluses and minus signs indicate the direction of change within the perturbation experiment relative to the baseline experiment. NA indicates no significant affect on a given variable.

**Parameter**	Utqiaġvik	Toolik	Eight Mile Lake	Delta Junction
Air temp. (°C)	+ 1.3	+ 1.5	+ 0.8	+ 1.5
Soil temp. (°C)	+ 0.85	+ 1.1	+ 0.3	+ 0.9
Volumetric water content (m^3 m^{-3})	- 0.3	- 0.3	- 0.3	- 0.6
Snowpack (m)	NA	NA	NA	NA
Ammonium (g m^{-2})	NA	NA	NA	+ 0.02
Nitrate (g m^{-2})	NA	NA	NA	NA
Microbial Biomass-N (g m^{-2})	+ 0.15	+ 0.17	+ 0.11	+ 8
Phosphate (g m^{-2})	- 0.22	NA	- 0.75	+ 0.12
Microbial Biomass-P (g m^{-2})	+ 0.22	NA	+ 0.15	+ 0.1

*maximum change in volumetric water content over the 10 year period.

Supplementary Table 3. Simulated site characteristics prior to the onset of perturbation (year 2000), and the ten-year averages for years 2050 and 2100 for (a) RCP.8.5-FF and (b) RCP.8.5-T. Values in the table represent the mean value and the standard deviation across ten years of daily output. For volumetric soil content output is given for surface soils (0.03 m) and subsurface (1.7 m) soils. **Ecosys* simulates microbial activity under winter snowpack, and the final column in each table provides the percentage increase in winter respiration over the baseline value derived from the 2000-2010 period (and the average magnitude in heterotrophic CO2 production over the winter period in gC m^{-2}) ± standard error.

(a) RCP.8.5-FF

Site	Year	MAT (°C)	MAP (mm)	Volumetric water content $(m^3\ m^{-3})$	Snowpack (m)	ALD (m)	% increase in winter resp.*
Utqiaġvik	2000 - 2010	-8.7 ± 3.1[1]	76 ± 18.3[1]	0.1 ± 0.15/ 0.08 ± 0.005	0.24 ± 0.08	-0.25 ± 0.12[1]	(0.04 ± 0.001)
	2045 - 2055	-3.4 ± 3.9	102.9 ± 22.1	0.16 ± 0.18/ 0.08 ± 0.005	0.24 ± 0.09	-0.34 ± 0.12	150 % (0.1 ± 0.00)
	2090 - 2100	1.3 ± 5.1	124.2 ± 26.9	0.21 ± 0.2/ 0.08 ± 0.005	0.22 ± 0.1	-0.52 ± 0.4	350 % (0.18 ± 0.00)
Toolik	2000 - 2010	-7.2 ± 3.6[2]	150.5 ± 42.8[2]	0.09 ± 0.14/ 0.09 ± 0.008	0.29 ± 0.07	-0.22 ± 0.09[2]	(0.1 ± 0.01)
	2045 - 2055	0.9 ± 4.2	180 ± 48.7	0.16 ± 0.14 / 0.1 ± 0.008	0.68 ± 0.08	-0.29 ± 0.33	120 % (0.22 ± 0.03)
	2090 - 2100	5.9 ± 4.8	205.3 ± 52.8	0.26 ± 0.24/ 0.15 ± 0.009	0.72 ± 0.07	-0.6 ± 0.3	300 % (0.4 ± 0.03)
Eight Mile Lake	2000 - 2010	-1.85 ± 3.8[3]	198 ± 47[3]	0.12 ± 0.11/ 0.14 ± 0.01	0.37 ± 0.07	-0.52 ± 0.37[3]	(0.2 ± 0.01)
	2045 - 2055	3.9 ± 3.8	232 ± 55.2	0.16 ± 0.14/ 0.34 ± 0.04	0.6 ± 0.1	-0.98 ± 0.4	140 % (0.48 ± 0.01)
	2090 - 2100	9.8 ± 4.2	260 ± 67.6	0.17 ± 0.13/ 0.42 ± 0.02	0.66 ± 0.06	-1.6 ± 0.5	300 % (0.8 ± 0.01)
Delta Junction	2000 - 2010	-1.7 ± 2.6[4]	257.6 ± 73.7[4]	0.17 ± 0.07/ 0.21 ± 0.01	0.8 ± 0.09	N/A	(0.5 ± 0.04)
	2045 - 2055	4.9 ± 3.2	326 ± 87.4	0.2 ± 0.07/ 0.3 ± 0.01	0.78 ± 0.07	N/A	240 % (1.7 ± 0.08)
	2090 - 2100	10.2 ± 4.4	348 ± 94.3	0.23 ± 0.06/ 0.3 ± 0.01	0.8 ± 0.09	N/A	540 % (3.2 ± 0.1)

(b) RCP.8.5-T.

Site	Year	MAT (°C)	MAP (mm)	Volumetric water content (m^3 m^{-3})	Snowpack (m)	ALD (m)	% increase in winter resp.
Utqiaġvik	2000 - 2010	-8.7 ± 3.1[1]	76 ± 36.2[1]	0.11 ± 0.15/ 0.08 ± 0.005	0.24 ± 0.08	-0.25 ± 0.12[1]	(0.04 ± 0.001)
	2045 - 2055	-3.4 ± 3.9	79.3 ± 37.8	0.13 ± 0.17/ 0.08 ± 0.005	0.21 ± 0.08	-0.39 ± 0.12	100 % (0.08 ± 0.00)
	2090 - 2100	1.3 ± 5.1	80.3 ± 37.3	0.17 ± 0.19/ 0.08 ± 0.005	0.17 ± 0.06	-0.52 ± 0.4	300 % (0.16 ± 0.00)
Toolik	2000 - 2010	-7.2 ± 3.6[2]	150.5 ± 57.8[2]	0.09 ± 0.14/ 0.09 ± 0.008	0.29 ± 0.07	-0.22 ± 0.09[2]	(0.1 ± 0.01)
	2045 - 2055	0.9 ± 4.2	155.9 ± 58	0.15 ± 0.18/ 0.10 ± 0.008	0.24 ± 0.08	–0.36 ± 0.09	100 % (0.2 ± 0.03)
	2090 - 2100	5.9 ± 4.8	152 ± 56.7	0.21 ± 0.21/ 0.15 ± 0.01	0.24 ± 0.06	-0.66 ± 0.36	200 % (0.3 ± 0.03)
Eight Mile Lake	2000 - 2010	-1.85 ± 3.8[3]	198 ± 40[3]	0.12 ± 0.11/ 0.13 ± 0.01	0.34 ± 0.08	-0.52 ± 0.37[3]	(0.2 ± 0.01)
	2045 - 2055	3.9 ± 3.8	211 ± 42.2	0.16 ± 0.13/ 0.36 ± 0.05	0.4 ± 0.08	-0.98 ± 0.4	115 % (0.43 ± 0.01)
	2090 - 2100	9.8 ± 4.2	204 ± 38.3	0.15 ± 0.12/ 0.41 ± 0.02	0.4 ± 0.06	-1.6 ± 0.5	260 % (0.72 ± 0.01)
Delta Junction	2000 - 2010	-1.7 ± 2.6[4]	257.6 ± 73.7[4]	0.17 ± 0.07/ 0.21 ± 0.01	0.8 ± 0.09	N/A	(0.5 ± 0.04)
	2045 - 2055	4.9 ± 3.2	293 ± 77.4	0.2 ± 0.07/ 0.3 ± 0.01	0.72 ± 0.07	N/A	160 % (1.3 ± 0.08)
	2090 - 2100	10.2 ± 4.4	294 ± 84	0.22 ± 0.06/ 0.3 ± 0.01	0.56 ± 0.09	N/A	260 % (1.8 ± 0.1)

Measured values:

[1]Utqiaġvik: MAT: -12.6 °C; MAP: 55 mm; ALD: 0.3 – 0.5 m (Oberbauer et al., 2007)

[2]Toolik: MAT: -8.6 °C; MAP: 180 mm (Oberbauer et al., 2007); ALD 0.33 – 0.45 m (Circumpolar active layer monitoring network)

[3]Eight Mile Lake: MAT: -2.3 ± 0.5 °C; MAP: 235 ± 31 mm; ALD: 0.5 m (Mauritiz et al., 2017)

[4]Delta Junction: MAT -2.1 °C; MAP: 290 mm (Mack et al., 2008)

Supplementary Table 4. Factors influencing SOC stocks over the multi-decadal simulations. The table reports data from Fig. S2, and provides the relative contribution of the most significant variables at each site, and each multi-decadal perturbation scenario (i.e., RCP8.5-fuly forced, and warming only), contributing to the change in SOC stocks. The transfer entropy approach provides the contribution of a given variable, within a network of all variables, to the variance of a dependent parameter (SOC stock). For example, for Utqiaġvik under scenario RCP8.5-FF, the SOC concentration has, approximately, 2.5x the influence on the changing SOC stock relative to the snowpack depth. For variables that repeat across multiple depths (i.e., soil moisture), the mean and standard deviation around the mean are provided.

Utqiaġvik

Variables	RCP8.5-FF	RCP8.5-T
SOC concentration	0.83	0.74
Soil moisture	0.78 ± 0.1	0.69
Soil temperature	0.75 ± 0.05	0.79
Active layer depth	0.72	0.7
Nutrient availability (N/ P)	0.7	0.5
Snowpack depth	0.31	0.42
Gross primary productivity	-	0.45

Toolik

Variables	RCP8.5-FF	RCP8.5-T
Soil temperature	0.9 ± 0.06	0.81
SOC concentration	0.83 ± 0.04	0.84
Snowpack depth	0.81	0.31
Nutrient availability (N/ P)	0.72	-
Soil moisture	0.71	0.82
Gross primary productivity	0.68	0.7
Net primary productivity	0.59	0.59
Ecosystem respiration	0.49	0.41

Eight Mile Lake

Variables	RCP8.5-FF	RCP8.5-T
Ecosystem respiration	0.56	0.81
Gross primary productivity	0.52	0.59
Net primary productivity	-	0.55
Nutrient availability (N/ P)	0.49	-
Soil temperature	0.49	0.51
SOC concentration	-	0.5

Delta Junction

Variables	RCP8.5-FF	RCP8.5-T
Gross primary productivity	0.8	0.76
Ecosystem respiration	0.7	0.6
SOC concentration	0.64 ± 0.1	0.58
Soil temperature	0.62 ± 0.05	0.66
Nutrient availability (N/ P)	0.54	0.61

Net primary productivity	0.53	0.5
Soil moisture	0.41	-
Snowpack depth	0.38	-

Supplementary Figures

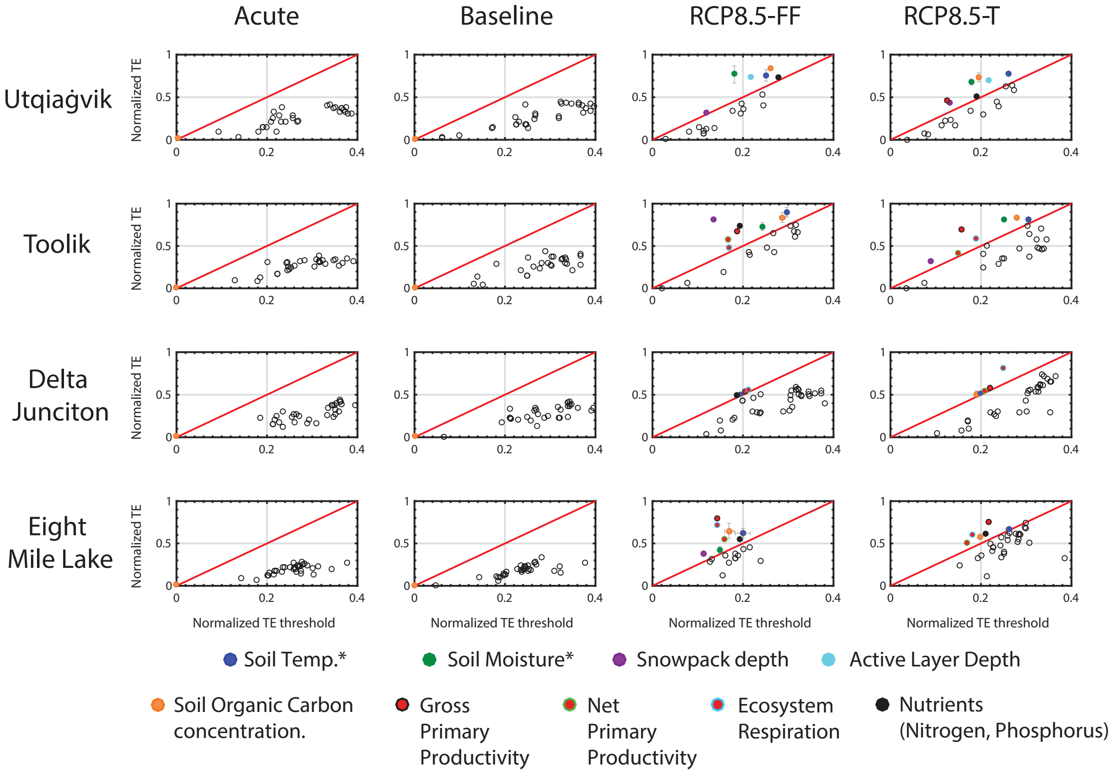

Supplementary Figure 1: Changes in vegetation and physical variables are predominantly responsible for modeled changes in SOC concentrations. The transfer entropy approach can identify out the most significant variables within a network that affect the SOC stock across the whole soil profile over time. Each panel of the figure represents either a short-term (acute/ baseline) or long-term (RCP8.5 fully forced or temperature-forcing only) simulation at each of the sites. The approach is described in detail in the methods section of the main text, but briefly, the figure can be read quantitatively. The time-series from which dependent and independent variables are take, is shuffled randomly, in order to dismantle causality, and calculate the transfer entropy (TE) between the shuffled time series. The random shuffle is repeated to calculate multiple TEs, and a 95 % confidence interval is derived from this random shuffle. The red line on the figure represents this 95 % confidence interval. Variables above this line are therefore statistically significant variables. The x-axis represents the transfer entropy significance threshold, and the normalized transfer entropy (on the y-axis) provides the contribution of a given variable within the network, to the change in the dependent variable (SOC stock). For ease of interpretation the various factors are colored by the broad category they fall into. For each site, the relative contribution of the most significant variables towards explaining the variance in SOC stock is given in table S5.

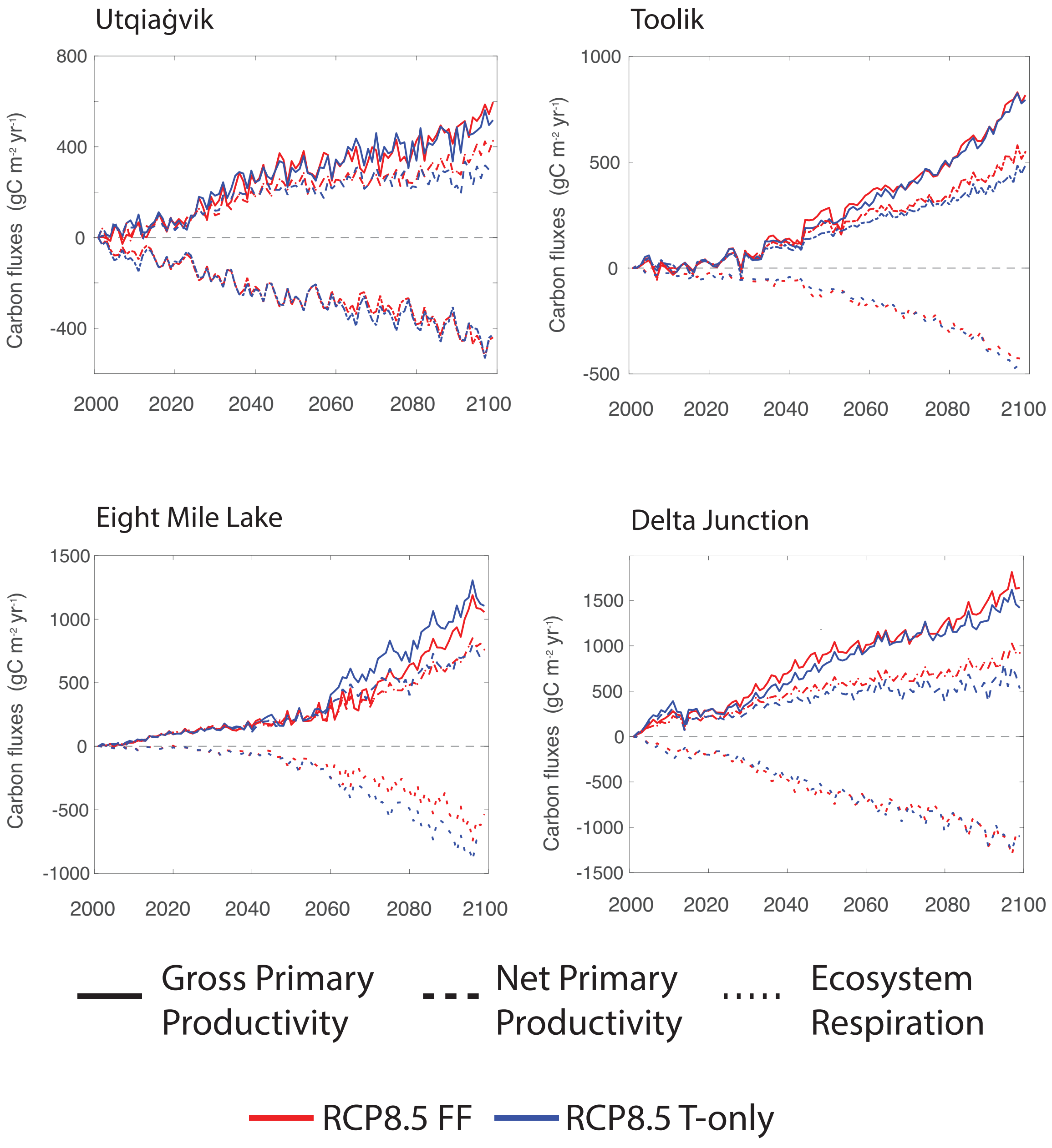

Supplementary Figure 2: Carbon fluxes increase over time under RCP8.5-FF and -T. Figure shows the change in Gross Primary Productivity (GPP), Net Primary Productivity (NPP), and Ecosystem respiration (R_{eco}) under the two RCP8.5 scenarios. Values are calculated as an annual mean, and output is normalized to baseline simulations. The figure is plotted related to *ecosys* carbon conventions where carbon leaving the ecosystem (via respiration) is plotted on a negative axis. Carbon being fixed into an ecosystem (via NPP or GPP) is plotted on a positive access. The inserts give Net Ecosystem Exchange (g C m^{-2} yr^{-1}) for each site.

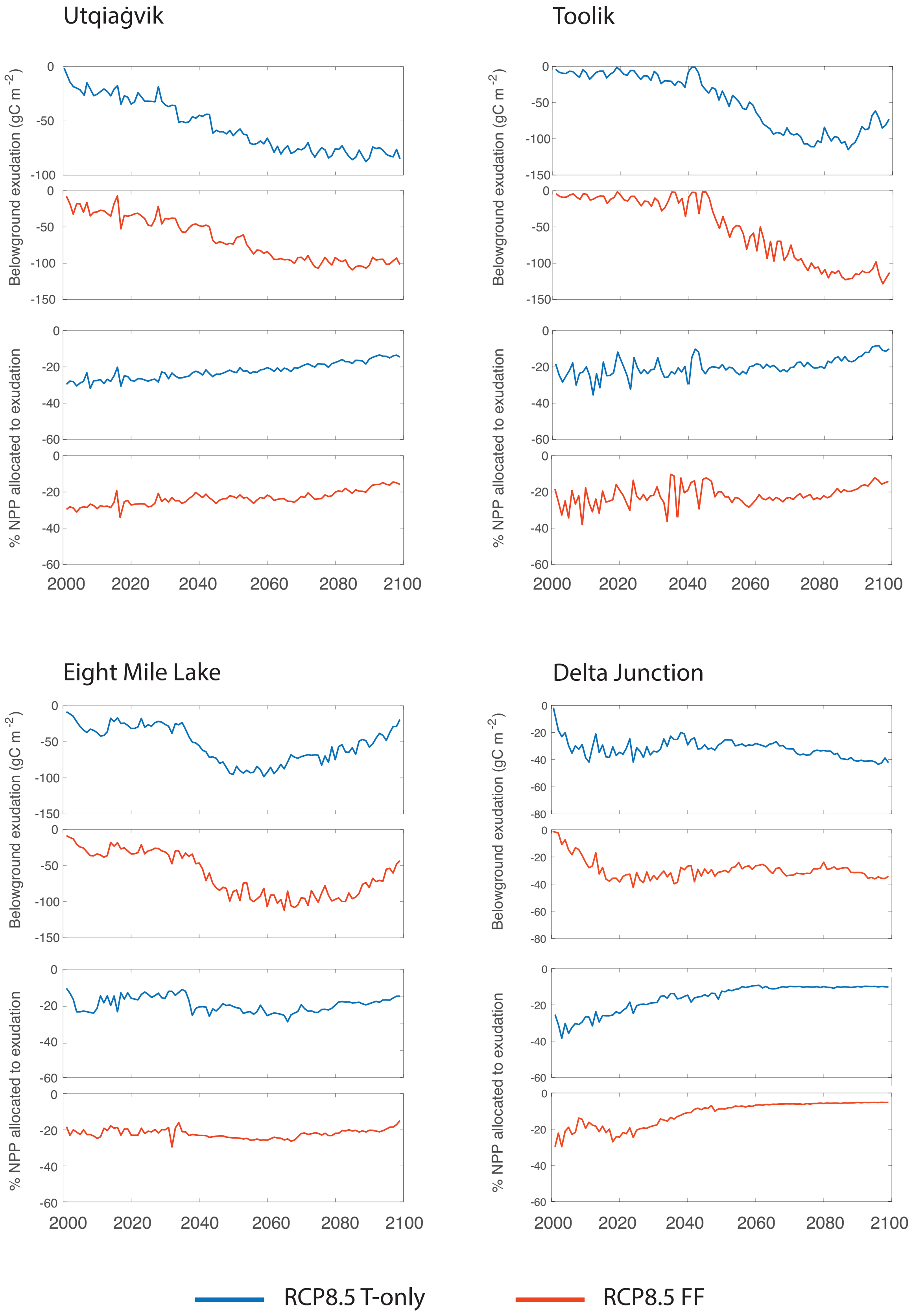

Utqiaġvik
Toolik
Eight Mile Lake
Delta Junction
Belowground exudation (gC m -2)
% NPP allocated to exudation
2000
2020
2040
2060
2080
2100
RCP8.5 T-only
RCP8.5 FF

Supplementary Figure 3: Belowground exudation: (a) Magnitude of carbon allocated belowground (g m-2) under warming only and full climate forcing scenarios over time. (b) Belowground exudation as a percentage of net primary productivity.

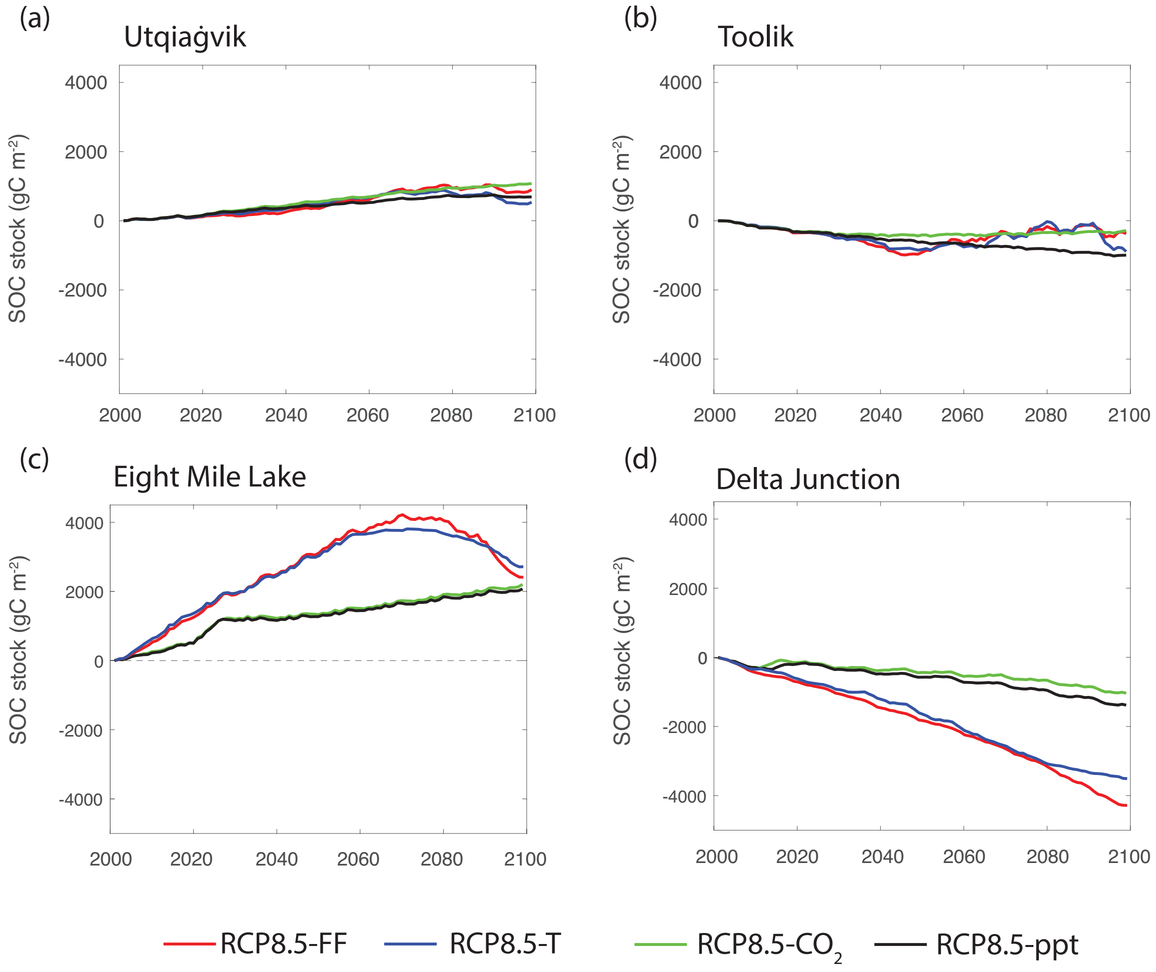

Supplementary Figure 4: Whole soil profile SOC stocks in response to full and single forcing factors. Each panel shows the site-specific SOC stock trajectories under four different forcing scenarios (The RCP8.5-FF and RCP8.5-T simulations are the same as those shown in Fig. 2b). Fully forced and warming only are the same as that described above. Elevated CO_2, and precipitation independently vary these factors while keeping all other variables (i.e., temperature, relative humidity, solar radiation) the same. Note, within the Toolik simulations the warming and precipitation scenarios show the same trajectory.

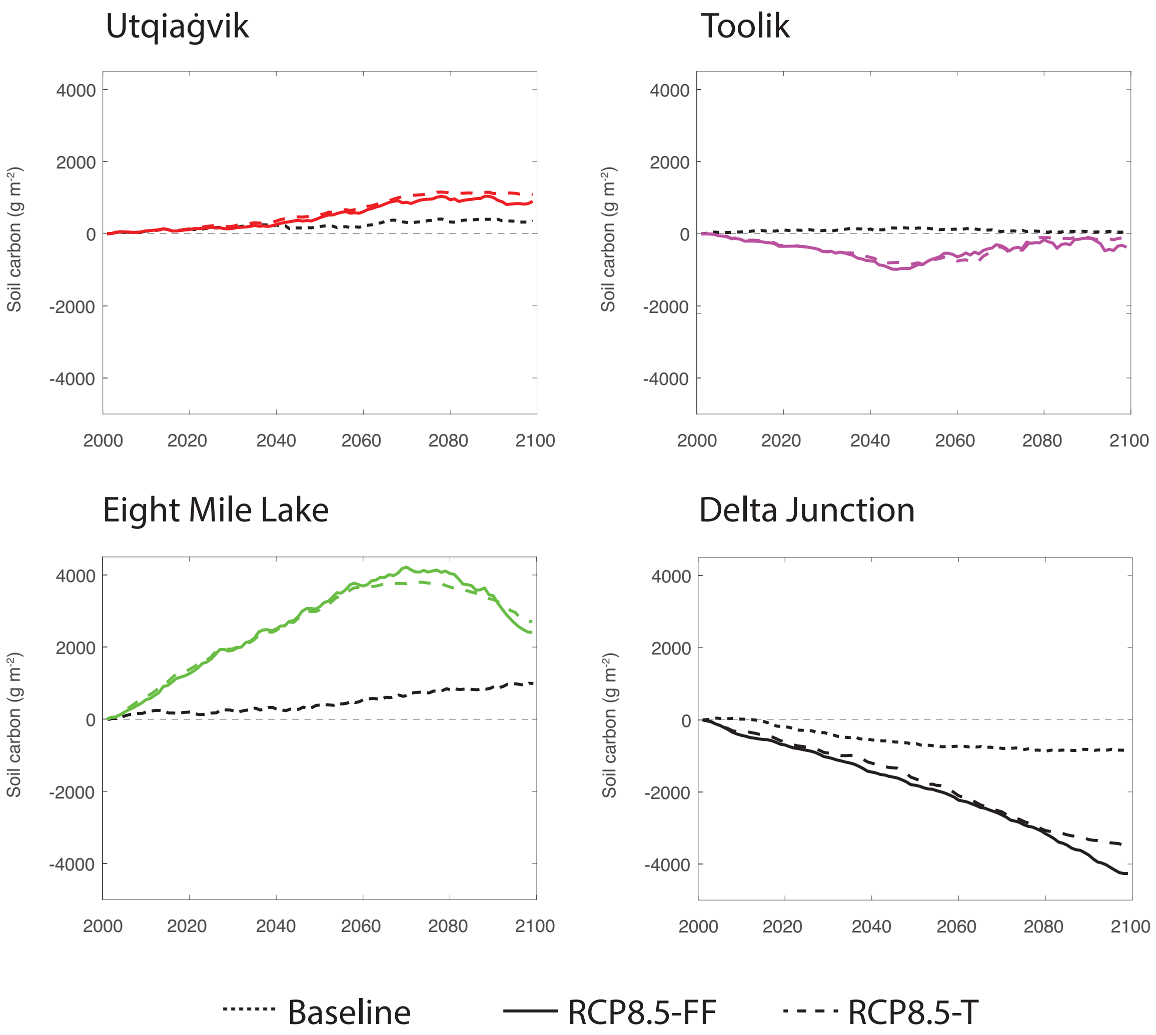

Supplementary Figure 5: Model perturbation shapes the ecosystem response and soil carbon cycle: Whole profile soil carbon trajectories under perturbation and continued baseline simulations. Baseline runs were also restarted at the year 2000, in keeping with the perturbation simulations, and continued out to 2100.

Supplementary references

1. Walker, M. D. *et al.* Plant community responses to experimental warming across the tundra biome. 5.

2. Sistla, S. A. *et al.* Long-term warming restructures Arctic tundra without changing net soil carbon storage. *Nature* **497**, 615–618 (2013).

3. Schuur, E. A. G. *et al.* Climate change and the permafrost carbon feedback. *Nature* **520**, 171–179 (2015).

4. Bintanja, R. & Andry, O. Towards a rain-dominated Arctic. *Nat. Clim. Change* **7**, 263–267 (2017).

5. Bintanja, R. & Selten, F. M. Future increases in Arctic precipitation linked to local evaporation and sea-ice retreat. *Nature* **509**, 479–482 (2014).

6. Koven, C. D., Lawrence, D. M. & Riley, W. J. Permafrost carbon−climate feedback is sensitive to deep soil carbon decomposability but not deep soil nitrogen dynamics. *Proc. Natl. Acad. Sci.* **112**, 3752–3757 (2015).

7. Prevéy, J. *et al.* Greater temperature sensitivity of plant phenology at colder sites: implications for convergence across northern latitudes. *Glob. Change Biol.* **23**, 2660–2671 (2017).

8. Press, M. C., Potter, J. A., Burke, M. J. W., Callaghan, T. V. & Lee, J. A. Responses of a subarctic dwarf shrub heath community to simulated environmental change. *J. Ecol.* **86**, 315–327 (1998).

9. McGraw, J. B. Experimental Ecology of Dryas octopetala Ecotypes. III. Environmental Factors and Plant Growth. *Arct. Alp. Res.* **17**, 229 (1985).

10. Keuper, F. *et al.* Tundra in the Rain: Differential Vegetation Responses to Three Years of Experimentally Doubled Summer Precipitation in Siberian Shrub and Swedish Bog Tundra. *AMBIO* **41**, 269–280 (2012).

11. Terrer, C. *et al.* Ecosystem responses to elevated CO_2 governed by plant-soil interactions and the cost of nitrogen acquisition. *New Phytol.* **217**, 507–522 (2018).

12. Grulke, N. E., Riechers, G. H., Oechel, W. C., Hjelm, U. & Jaeger, C. Carbon balance in tussock tundra under ambient and elevated atmospheric CO2. *Oecologia* **83**, 485–494 (1990).

13. Sigurdsson, B. D., Medhurst, J. L., Wallin, G., Eggertsson, O. & Linder, S. Growth of mature boreal Norway spruce was not affected by elevated [CO2] and/or air temperature unless nutrient availability was improved. *Tree Physiol.* **33**, 1192–1205 (2013).
